# The GGH/HuR Complex Binds and Stabilizes mRNAs to Maintain Tumor Cell Cycle and DNA Replication

**DOI:** 10.1002/advs.202500838

**Published:** 2025-08-19

**Authors:** Yu Li, Xinrui Li, Yuhui Du, Sijie Chen, Xiaoniu He, Zhangrong Xie, Zhiqing Zhou, Huijie Zhao, Xiaofei Zeng, Guoan Chen

**Affiliations:** ^1^ Department of Critical Care Medicine Fourth People's Hospital School of Medicine Tongji University Shanghai 200434 China; ^2^ Department of Human Cell Biology and Genetics Joint Laboratory of Guangdong‐Hong Kong Universities for Vascular Homeostasis and Diseases SUSTech Homeostatic Medicine Institute School of Medicine Southern University of Science and Technology Shenzhen Guangdong 518055 China; ^3^ State Key Laboratory for Conservation and Utilization of Bio‐resource in Yunnan and School of Ecology and Environmental Science Yunnan University Kunming 650500 China; ^4^ Shanxi Bethune Hospital Shanxi Academy of Medical Sciences Third Hospital of Shanxi Medical University Tongji Shanxi Hospital Taiyuan 030032 China; ^5^ H&H Group H&H Research China Research and Innovation Center Guangzhou 510700 China; ^6^ Department of Oncology Sun Yat‐sen Memorial Hospital Sun Yat‐sen University Guangzhou 510120 China; ^7^ National Key Laboratory for Tropical Crop Breeding Shenzhen Branch Guangdong Laboratory for Lingnan Modern Agriculture Genome Analysis Laboratory of the Ministry of Agriculture Agricultural Genomics Institute at Shenzhen Chinese Academy of Agricultural Sciences Shenzhen 518120 China

**Keywords:** GGH, HuR, RNA‐binding protein, mRNA stability, lung cancer

## Abstract

GGH (Gamma‐glutamyl hydrolase) is a folate metabolism enzyme that hydrolyzes intracellular polyglutamylated folates and is highly expressed in various cancers. It remains unclear whether GGH functions as an oncogene and its underlying mechanisms in tumor progression. Here, it is reported that GGH silencing inhibited the growth of lung cancer cells in vivo and in vitro. The oncogenic function of GGH relied on its non‐canonical role as a novel RNA‐binding protein, which maintained the cell cycle and DNA replication by stabilizing target mRNAs. Furthermore, GGH bound to the GC‐rich motif in the 5′ untranslated region of mRNAs, such as CDC6 and CCND1. Additionally, GGH directly interacts with HuR (Human Antigen R), a well‐characterized RNA‐binding protein critical for mRNA stability in cancer. GGH, HuR, and their mRNA targets formed a ternary complex, which may facilitate the induction of a circular mRNA conformation, potentially enhancing RNA stability. Finally, it is found that GGH is highly expressed in lung cancer tissues, and its elevated expression correlates with worse patient survival in lung cancer. This discovery offered novel insights and identified potential therapeutic targets for the prevention and treatment of lung cancer.

## Graphical abstract

Despite its canonical role in inhibiting DNA synthesis, GGH promotes tumor growth as a novel RNA‐binding protein. GGH binds GC‐rich 5′UTRs (e.g., CDC6/CCND1), recruits HuR to form a ternary complex that stabilizes mRNA via circular conformation, fueling DNA replication and the cell cycle. Targeting this axis suppresses NSCLC progression.

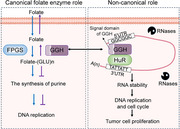

## 1 Introduction

The eukaryotic cell cycle is essential for life, comprising two main events: DNA replication (S phase) and segregation of replicated DNA (M phase).^[^ ^1^ ^]^ A complex network regulates this cycle to ensure precise timing and favorable conditions, maintaining tissue health and preventing abnormal cell growth.^[^ ^2^ ^]^ Following the pre‐replicative G1 phase, the G1/S checkpoint assesses DNA quality and environmental factors before allowing the cell to proceed to the S phase.^[^ ^3^ ^]^ The relationship between DNA replication and cell cycle regulation is closely linked through the G1/S checkpoint. DNA replication occurs in the S phase and requires passing the G1/S checkpoint. The pre‐replication complex must form early in G1 to initiate replication and enter the S phase.^[^ ^4^ ^]^ Cells in G1 can also enter a non‐proliferative, quiescent state (G0), where most adult body cells reside. However, cancer cells are primarily impaired in their ability to exit the cell cycle, causing continuous division and uncontrolled proliferation.^[^ ^5^ ^]^ Therefore, tumors are often regarded as a cell cycle‐related disease.^[^ ^6^ ^]^

GGH (Gamma‐glutamyl hydrolase) is a folate metabolism enzyme that reduces intracellular polyglutamylated folate levels by hydrolyzing them into transportable monoglutamates.^[^ ^7^ ^]^ Polyglutamylated folate, an active form of folate, is integral to one‐carbon transfer processes, thereby contributing to purine biosynthesis and DNA synthesis.^[^ ^8^ ^]^ It not only provides essential substrates for DNA replication, but also acts as a cofactor for folate‐dependent enzymes, facilitating cell cycle regulation and proliferation.^[^ ^9^ ^]^ In tumor cells, the depletion of folate, particularly in its polyglutamylated form, disrupts DNA synthesis and consequently inhibits tumor growth.^[^ ^10^ ^]^ This concept underlies the use of antifolate drugs, such as methotrexate (MTX) and 5‐fluorouracil (5FU), in cancer chemotherapy.^[^ ^11^ ^]^ Several studies have found that GGH is highly expressed in multiple cancers and promotes cell cycle progression and proliferation.^[^ ^12^ ^]^ However, the exact mechanism underlying this phenomenon remains unclear, with the folate hydrolase activity of GGH only being speculated as a potential cause. In fact, the classic enzymatic function of GGH reduces intracellular polyglutamylated folate levels and inhibits DNA synthesis, which is contrary to the observed promotion of cell cycle progression. Therefore, it is crucial to uncover the true underlying mechanism by which GGH promotes cell cycle in tumors.

RNA‐binding proteins (RBPs) are diverse proteins that interact with RNAs to form ribonucleoprotein complexes, influencing RNA fate and regulating gene expression aspects like RNA stability, splicing, and translation.^[^ ^13^ ^]^ These functions impact tumor cell progression, affecting processes such as the cell cycle, proliferation, apoptosis, and migration.^[^ ^14^ ^]^ Hu Antigen R (HuR, also known as ELAVL1) is a member of the ELAVL family that is one of the most well‐studied RNA‐binding proteins, known for binding to AU‐rich elements (AREs) in the 3' UTR of target RNAs, thereby stabilizing them.^[^ ^15^ ^]^ HuR exerts its oncogenic functions by binding to and stabilizing some key RNAs, particularly those involved in the regulation of the cell cycle and DNA replication, such as CDC6, CCND1, MYC, FOS, and p21.^[^ ^16^ ^]^

Lung cancer has the highest incidence and mortality rate in the world, making it a significant public health concern.^[^ ^17^ ^]^ Lung cancer is primarily classified into non‐small cell lung cancer (NSCLC) and small cell lung cancer (SCLC), with NSCLC accounting for ≈80–85% of all lung cancer cases.^[^ ^18^ ^]^ Meanwhile, lung adenocarcinoma (LUAD) is the main subtype of NSCLC, accounting for ≈60% of all NSCLC cases. Although several anti‐cancer strategies like surgery, chemotherapy, and irradiation are used, lung cancer remains difficult to treat due to its complexity and drug resistance.^[^ ^19^ ^]^ Therefore, there is an urgent need for a better understanding and targeted therapies to cure or manage lung cancer.

In this study, we unexpectedly identified a non‐canonical function of GGH as a novel RBP that maintained the cell cycle and DNA replication by stabilizing its mRNA targets. This non‐canonical function of GGH was primarily observed in NSCLC compared to normal lung cells and tissues. GGH was also highly expressed in lung cancer tissues and its higher expression was correlated to poor patient survival in lung cancer. GGH deficiency suppressed cell growth of NSCLC both in vitro and in vivo. Hence, GGH functioned as an oncogene that relied on its non‐canonical role. In addition, GGH also acted as a novel HuR binding partner during the tumor process. GGH, HuR, and mRNA targets formed a ternary complex where each component enhanced the binding interactions of the other two. Collectively, GGH, as a novel RBP, formed a ternary complex with HuR and mRNA targets to stabilize RNAs, maintained the cell cycle and DNA replication, and promoted tumor growth in NSCLC. This discovery provided new insights and potential therapeutic targets for lung cancer prevention and treatment.

## 2 Results

### 2.1 GGH Knockdown Impairs the Cell Cycle and DNA Replication

**Figure 1:**
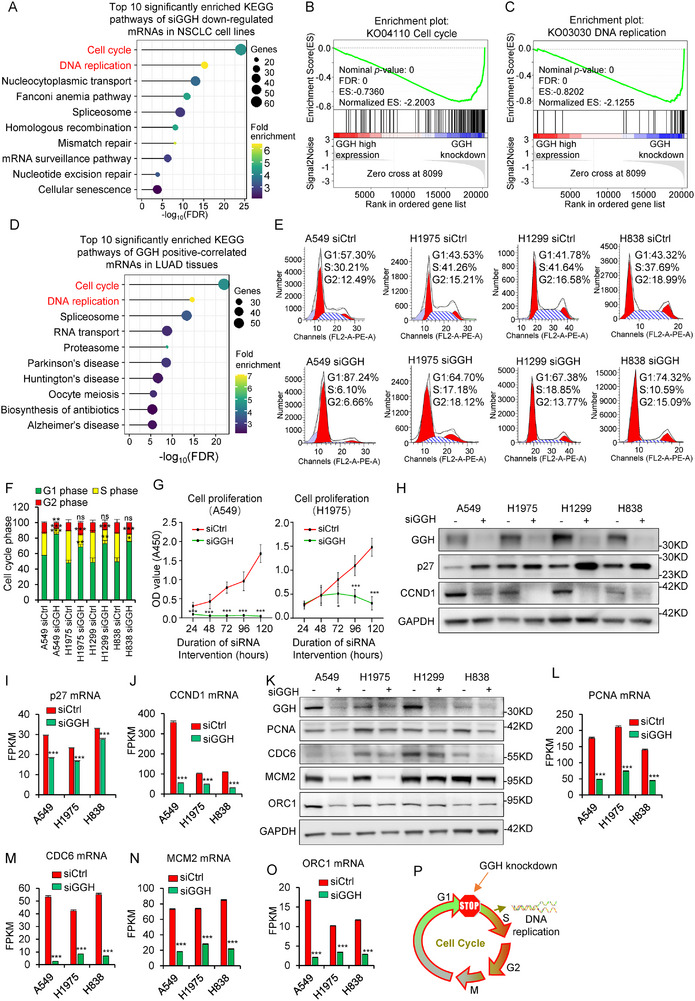
GGH knockdown impedes cell cycle and DNA replication. A) Enriched KEGG pathways of downregulated genes (mRNAs) upon GGH knockdown in NSCLC cell lines measured by RNA‐seq (2645 downregulated genes, at least 2/3 NSCLC cell lines, siGGH/siCtrl < 0.65). B,C) GSEA graphs for two enriched pathways, cell cycle and DNA replication. D) Enriched KEGG pathways of genes (mRNAs) positively correlated with GGH in NSCLC tissues (1726 genes, Pearson correlation, r≥ 0.3, n = 464). E) Representative images of the cell cycle analysis in NSCLC cell lines using flow cytometry. F) Cell cycle distribution of NSCLC cells with/without GGH knockdown. ^***^ indicated the ratio of G1/S phase, siGGH versus siCtrl group, *p*< 0.001. G) A549 and H1975 cell lines were transfected with siCtrl and siGGH and cultured for 24, 48, 72, 96, and 120 h. Cell proliferation was detected by CCK8 assay. Bars indicate SD, n = 6, ^*^, *p*< 0.05; ^**^, *p*< 0.01; ^***^, *p*< 0.001 H) Western blot of p27 and CCND1 after GGH knockdown for 72 h in NSCLC cell lines. I,J) mRNA levels of p27 and CCND1 measured by RNA‐seq in NSCLC cell lines. Bars indicate SD, n = 3, ^***^ *p*< 0.001. K) Western blot of PCNA, CDC6, MCM2, and ORC1 after GGH knockdown for 72 h in NSCLC cell lines. L–O) mRNA levels of PCNA, CDC6, MCM2 and ORC1 measured by RNA‐seq in NSCLC cell lines. Bars indicate SD, n = 3, ^***^ *p*< 0.001. P) Schematic of G1/S phase arrest and DNA replication inhibition upon GGH silencing. Statistical analysis in panels F, G, I, J and L‐O was performed by unpaired two‐tailed Student's t‐test.

GGH is highly expressed and promotes cell proliferation in several cancers, including glioma, uterine corpus endometrial carcinoma, breast cancer, and others.^[^ ^12^, ^20^ ^]^ However, the detailed mechanism underlying this effect has yet to be elucidated. To explore more functions and mechanisms of GGH in lung cancer progression, we performed RNA‐seq and DIA‐MS analysis upon GGH silencing by siRNAs in NSCLC cell lines. When GGH was silenced efficiently (Figure S1A–D, Supporting Information), cell cycle and DNA replication pathways exhibited the most significant down regulation in NSCLC cells identified from RNA‐seq (**Figure** **1**A–C). A decrease in mRNA expression was observed for the vast majority of genes involved in these two pathways (Figure S1E–H, Supporting Information). While in normal lung epithelial cells, the correlation between GGH knockdown and downregulation of cell cycle and DNA replication pathways was relatively weak (Figure S1I, Supporting Information). To confirm if GGH is related to cell cycle and DNA replication in human lung cancer tissues, we performed Pearson correlation analysis between GGH mRNA expression and other genes in LUAD tissues (RNA‐seq data from three public datasets: the UM cohort,^[^ ^21^ ^]^ the Seo cohort,^[^ ^22^ ^]^ and TCGA‐LUAD) and found that genes positively correlated with GGH were also strongly involved in the cell cycle and DNA replication pathways (Figure 1D). This correlation was weaker in adjacent non‐cancerous tissues (Figure S1J, Supporting Information), suggesting that GGH was involved in the cell cycle and DNA replication in both cancer cell lines and tumor tissues. We performed flow cytometry analysis to evaluate whether siGGH affected the cell cycle. As shown in Figure 1E,F, and Figure S1K (Supporting Information), GGH knockdown arrested the cell cycle at the G1/S phase in NSCLC cells. In addition, compared with lung cancer cells, lung epithelial cells showed a weaker ability in G1/S phase arresting after siGGH transfection (Figure S1L–O, Supporting Information). Dysregulation of the cell cycle is a vital reason for tumor cell proliferation. To further explore whether GGH knockdown inhibited cell proliferation, we examined cell proliferation by CCK8 assay and found that silencing GGH suppressed cell proliferation in NSCLC cell lines (Figure 1G; Figure S1P,Q, Supporting Information). Consistent with G1/S phase arrest evaluated by flow cytometry, protein level of p27, a negative regulator of the cell cycle, was increased, whereas CCND1 (Cyclin D), a positive regulator of the cell cycle, was decreased in NSCLC cells after siGGH treatment (Figure 1H). Additionally, protein expressions of the important cell cycle‐related transcription factors E2F1, c‐MYC, and FOXM1 decreased upon GGH knockdown (Figure S1R, Supporting Information). The mRNA levels of these cell cycle marker genes all declined (Figure 1I,J; Figure S1S–U, Supporting Information). Meanwhile, protein levels of PCNA (a DNA polymerase sliding clamp) and key pre‐replication complex (preRC) components–CDC6 and MCM2 involved in DNA replication initiation and elongation were reduced (Figure 1K). ORC1 protein expression was reduced only in A549 and H1975 cells, showing no significant alteration in H838 and H1299 cells (Figure 1K). The mRNA levels of PCNA, CDC6, MCM2, and MCM3‐7 components significantly declined in all cell lines, as did ORC1 mRNA (Figure 1L–O; Figure S1V–Z, Supporting Information). These results demonstrated that silencing of GGH can cause cell cycle arrest and impede DNA replication, especially in NSCLC cells (Figure 1P). This effect may be due to the reduced mRNA expression of specific genes in these two pathways.

### 2.2 GGH Silencing Diminishes Cell Cycle and DNA Replication Independent of Folate Metabolism Pathways

**Figure 2:**
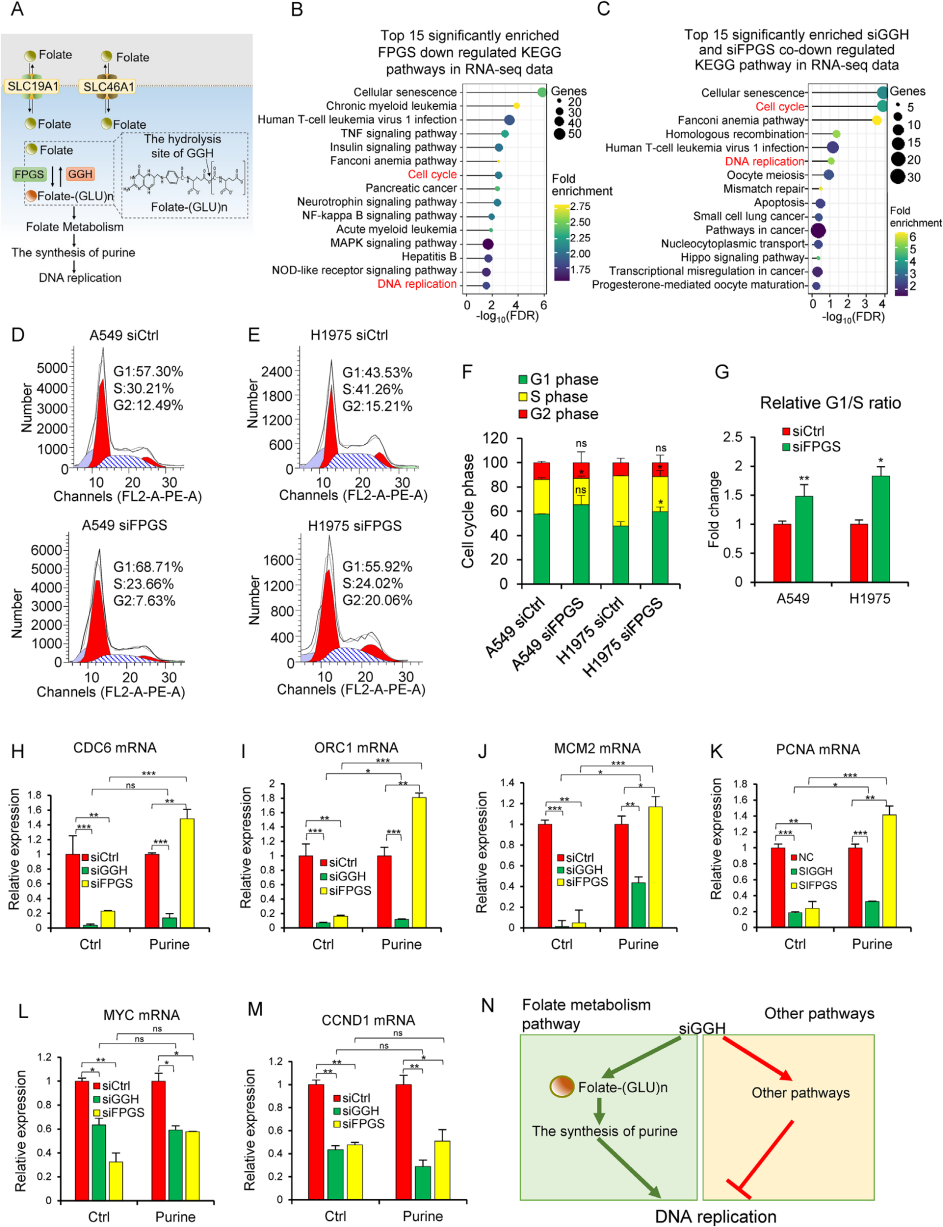
GGH knockdown diminishes DNA replication and cell cycle independent of folate metabolism. A) Schematic of the role of GGH and FPGS in folate metabolism and DNA replication (green circles marked monoglutamated folate, and orange circles marked polyglutamylated folate). B) Enriched KEGG pathways of downregulated genes (mRNAs) upon FPGS knockdown in NSCLC cell lines measured by RNA‐seq (2604 downregulated genes, A549 and H1975 cell lines, siFPGS/siCtrl < 0.65). C) Enriched KEGG pathways of the overlapping of downregulated genes (mRNAs) upon FPGS and GGH knockdown in NSCLC cell lines (737 co‐downregulated genes, siFPGS/siCtrl<0.65 and siGGH/siCtrl< 0.65). D,E) Representative images of the cell cycle analysis in A549 and H1975 using flow cytometry. F) Cell cycle distribution of A549 and H1975 cell lines with/without FPGS knockdown. Values were mean ± SD from n =3 independent experiments, siFPGS vs. siCtrl group, ^*^, *p*< 0.05. G) The ratio of the G1/S phase upon FPGS knockdown in A549 and H1975 cells. Values were mean ± SD from n =3 independent experiments, ^**^, *p*<0.01, ^*^, *p*<0.05. H–K) qRT‐PCR was used to test the mRNA expression of DNA replication initiation‐related genes after siGGH or siFPGS transfection with/without purine treatment. Values were mean ± SD from n =3 independent experiments. ^*^, *p*< 0.05, ^**^, *p*< 0.01, ^***^, *p* < 0.001. L,M) The mRNA levels of cell cycle‐related genes were measured by RT‐PCR after siGGH or siFPGS transfection with/without purine (0.4 mm) treatment. N) Schematic of potential pathways through which GGH influenced DNA replication. Statistical analysis in panels F and G was performed using unpaired two‐tailed Student's t‐test, whereas that in panels H–M was performed using one‐way ANOVA with multiple comparisons.

GGH is an important folate metabolism enzyme and folates are crucial for the biosynthesis of nucleotides.^[^ ^23^ ^]^ Intracellular folate and its active form, polyglutamylated folate, are involved in one‐carbon transfer, contributing to purine production and supporting DNA synthesis.^[^ ^24^ ^]^ GGH and folylpolyglutamate synthetase (FPGS) have opposing functions regarding DNA synthesis. GGH hydrolyzes polyglutamated folates, thereby suppressing intracellular folate metabolism and reducing purine biosynthesis, which ultimately inhibits DNA synthesis. Conversely, FPGS catalyzes the synthesis of polyglutamated folates, promoting folate‐dependent nucleotide production and DNA synthesis (**Figure** **2**A).^[^ ^25^ ^]^ We aimed to determine if FPGS has a function opposite to GGH in regulating the cell cycle and DNA replication, and how both genes influence these processes depending on folate metabolism.

To confirm whether FPGS affects DNA replication and cell cycle, we knocked down FPGS and performed RNA‐seq analysis (Figure S2A,B, Supporting Information). As expected, the DNA replication and cell cycle pathways were affected by the down regulated genes of FPGS silencing in A549 and H1975 cell lines (Figure 2B), although they were not at the top pathways as shown in GGH knockdown in Figure 1A, suggesting FPGS had other functions such as cellular senescence, TNF signaling, and NF‐kB, etc. Furthermore, the overlapped down‐regulated genes by siGGH and siFPGS were also involved in DNA replication and cell cycle pathways in NSCLC cell lines (Figure 2C), indicating these two genes have redundant roles in DNA replication and cell cycle. The cell cycle or DNA replication pathway was unaffected by overlapping genes under other conditions, such as siGGH‐down and siFPGS‐up, siGGH‐up and siFPGS‐down, or siGGH‐up and siFPGS‐up (Figure S2C–E, Supporting Information). In addition, FPGS knockdown arrested the cell cycle at the G1/S phase in both the NSCLC and lung epithelial cell lines, as analyzed by flow cytometry analysis (Figure 2D–G; Figure S2F–H, Supporting Information). These findings illustrated that GGH or FPGS knockdown could inhibit DNA replication and cell cycle pathways.

To further explore whether GGH and FPGS affect DNA replication and the cell cycle through folate metabolism, we performed rescue experiments by complementing folate metabolite purine. The mRNA levels of CDC6, ORC1, MCM2‐7, and PCNA were used as markers of DNA replication. We found that these mRNA expressions were down‐regulated following siFPGS or siGGH transfection, indicating DNA replication and cell cycle were impaired by FPGS knockdown or GGH knockdown (Figure 2H–K). Next, purine could fully reverse FPGS silencing‐induced down‐regulation of these 4 genes, whereas no such full reversal was observed with GGH silencing (Figure 2H–K; Figure S2I–M, Supporting Information). The results indicated that purine was fully capable of reversing the inhibition of DNA replication initiation and elongation induced by FPGS knockdown, suggesting FPGS affected DNA replication via folate metabolism. Although purine supplementation increased the transcript levels of some DNA replication‐related genes, it failed to fully reverse the DNA replication inhibition caused by GGH knockdown, unlike its ability to reverse FPGS knockdown‐induced inhibition. This suggests the involvement of mechanisms beyond folate metabolism. On the other hand, the downregulation of cell cycle markers ‐ MYC and CCND1 upon GGH and FPGS knockdown were not reversed by purine (Figure 2L,M). In addition, purine supplementation increased the S‐phase population in all groups (siCtrl, siGGH, and siFPGS), especially in siFPGS cells—reversing its G1/S arrest (Figure S2N,O, Supporting Information). These results indicated that FPGS affected DNA replication through the folate metabolism, especially purine metabolite. Cell cycle arrest upon FPGS knockdown is likely driven by DNA replication inhibition resulting from depletion of intracellular polyglutamated folates and subsequent purine deficiency. In contrast, GGH deficiency should result in polyglutamated folate accumulation to supply sufficient purine that promotes DNA replication and cell cycle, but we observed the opposite results, e.g. GGH knockdown impaired DNA replication. Hence, we speculated that the inhibitory effect of GGH knockdown on the two pathways may have other pathways, which was independent of folate metabolism (Figure 2N).

Besides that, other substrates attempted to reverse the suppression of DNA replication upon silencing of GGH or FPGS. This inhibitory effect of PCNA induced by silencing FPGS was fully reversed by purine but not by pyrimidine, which is not directly associated with folate metabolism (Figure S2P, Supporting Information). However, simultaneous supplementation of both purine and pyrimidine can elevate the mRNA levels of PCNA that were downregulated following GGH or FPGS knockdown (Figure S2P, Supporting Information). In addition, the supplementation of dNTP, an essential substrate in DNA replication, can also elevate the mRNA levels of DNA replication‐ and cell cycle‐related genes that were downregulated following the silencing of GGH or FPGS (Figure S2Q–V, Supporting Information). The dNTP supplementation partially restored mRNA levels of specific DNA replication and cell cycle genes (e.g., CDC6, ORC1, MCM2, PCNA, and MYC) in GGH and FPGS‐knockdown cells (Figure S2Q–V, Supporting Information). The results indicated that an adequate supply of DNA replication substrates that are not directly associated with folate metabolism could increase mRNA expression of DNA replication‐ and cell cycle‐related genes. These may result from an excessive amount of DNA, inducing increased nascent transcription. Overall, our data demonstrated that FPGS affected DNA replication through folate metabolism, while the role of GGH in the cell cycle and DNA replication was independent of folate metabolism.

### 2.3 GGH Binds with an RNA‐Binding Protein, HuR

**Figure 3:**
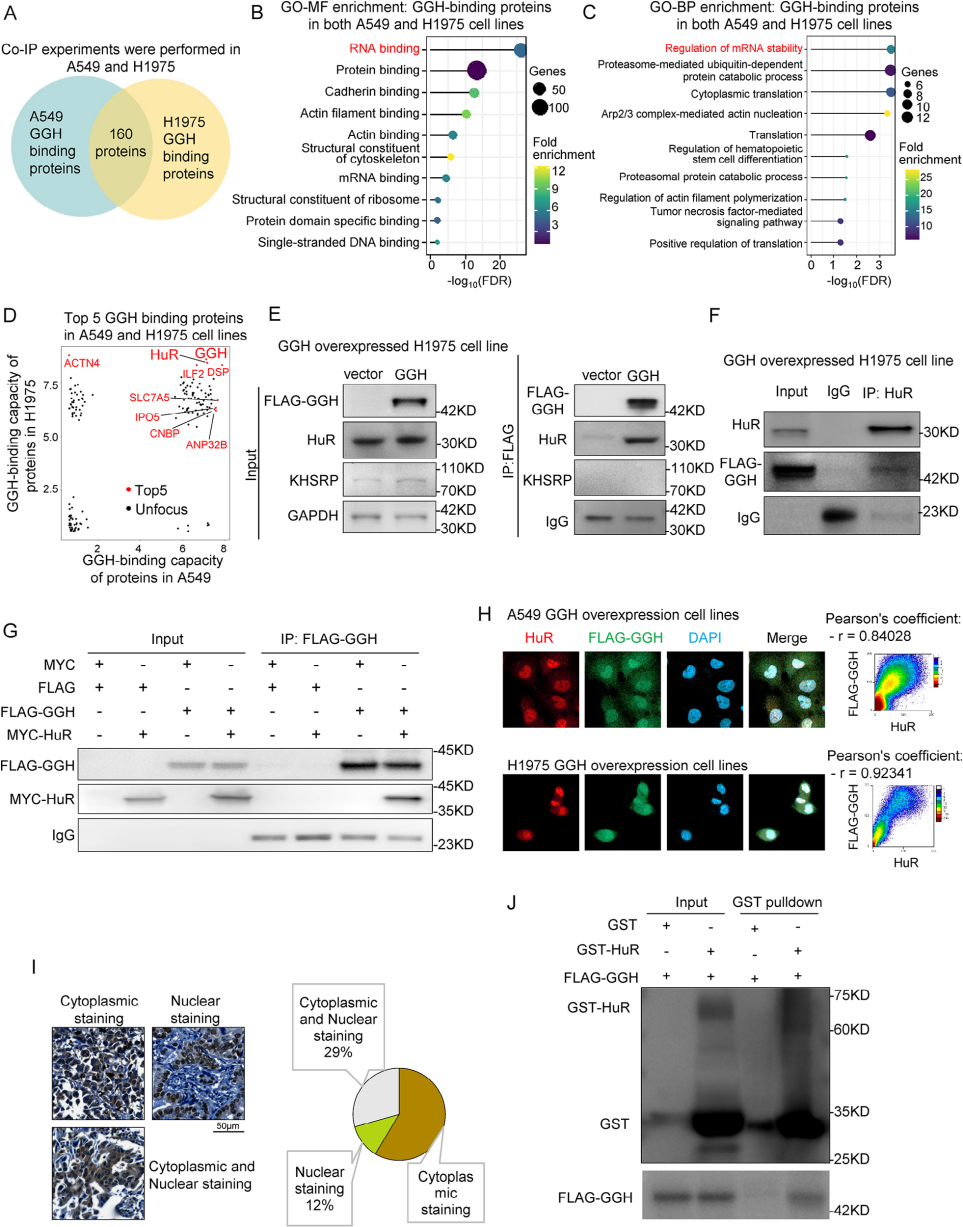
GGH binds with an RNA binding protein‐HuR. A) The Venn diagram showed the intersection of proteins binding with GGH in A549 and H1975 cell lines. Co‐IP MS experiments. B) GO‐MF enrichment analysis of GGH‐binding proteins. C) GO‐BP enrichment analysis of GGH‐binding proteins. D) Scatter plots of GGH‐binding capacity of proteins in A549 and H1975 cells, respectively. The top 5 proteins with the highest values (Log_10_((Area value of GGH over+1)/ (Area value of Vector +1)) among the proteins pulled down by GGH are marked in red. E) Detection of GGH‐associated candidate proteins by Co‐IP assays in GGH overexpressed H1975 cell. Both HuR and KHSRP are RNA‐binding proteins that bind to the 3' UTR AU‐rich element (ARE) region and may also interact with GGH. The GAPDH and immunoglobulin G (IgG) were used as controls. F) Co‐IP assays were performed with anti‐HuR antibodies, followed by immunoblotting with FLAG and HuR antibodies. Immunoglobulin G (IgG) was used as a negative control. (G) Co‐IP of exogenous GGH shown that GGH precipitates exogenous HuR in 293T cell. H) Protein co‐localization of GGH and HuR. Colocalization images (63X). I) Immunohistochemical staining revealed that GGH was located in different subcellular compartments. The left was representative GGH IHC images of different subcellular localizations (scale bar = 50 µm). The right was a pie chart that represents the percentage distribution of GGH across different subcellular compartments. NSCLC tissue microarray (TMA) contained 75 individual tumor samples. J) GST‐pulldown assays of GST–HuR pulling down GGH.

To explore bona fide mechanisms underlying how GGH affected the DNA replication and cell cycle, we first performed a Co‐IP assay followed by a quantitative mass spectrometry analysis to identify a possible FLAG‐GGH protein interactome in A549 and H1975 cells (**Figure** **3**A). Intriguingly, the most enriched protein category that interacts with GGH was RNA‐binding proteins, especially those that regulate mRNA stability (Figure 3B,C). Interestingly, HuR was one of the most strongly interacting proteins to bind with GGH in A549 and H1975 cell lines (Figure 3D). HuR is known to be a classical RNA‐binding protein and stabilizes ARE‐containing mRNAs.^[^ ^26^ ^]^ Therefore, we verified the interaction between GGH and HuR by Co‐IP and Western blot experiments. In H1975 cells, we confirmed that exogenously expressed GGH could pull down endogenous HuR, but not KHSRP, an RNA‐binding protein that also binds to the 3′ UTR AU‐rich element (ARE) region (Figure 3E). Inversely, endogenously expressed HuR could pull down exogenously expressed GGH in H1975 cell as well (Figure 3F). Exogenously expressed GGH and HuR were capable of mutual Co‐IP in 293T cells (Figure 3G; Figure S3A, Supporting Information). The immunofluorescent staining experiment also indicated that GGH protein was co‐localized with HuR protein (Figure 3H). Next, to further examine the subcellular localization of the GGH and HuR proteins, we performed GGH immunohistochemistry of NSCLC tissues. The GGH protein was localized in both the nucleus and the cytosol (Figure 3I). Besides this, Western blot assay also confirmed the same subcellular localization of the GGH (Figure S3B, Supporting Information). HuR is reported to be abundantly localized to the nucleus but shuttles between the nucleus and cytoplasm.^[^ ^27^ ^]^ We found that HuR was predominantly localized in the nucleus, with only a minimal fraction presented in the cytoplasm (Figure 3H). By GST‐pulldown in vitro assays, we found that GST–HuR, but not GST alone, could effectively pulldown translated GGH (Figure 3J). Those results indicated that GGH could bind with HuR directly.

GGH protein comprises two domains, signal domain (amino acids1‐24) and peptidase domain (amino acids 25‐318) (Figure S3C, Supporting Information). To further explore the binding site of GGH, we constructed the GGH domain 1 and 2 plasmids and performed Co‐IP assays (Figure S3D,E, Supporting Information). Both GGH domain 1 and domain 2 could reciprocally CO‐IP with exogenously expressed HuR in 293T cells (Figure S3F–I, Supporting Information). We next tried to conduct a molecular docking analysis to identify the binding sites of GGH and HuR by AlphaFold 3. As shown in Figure S3J‐L (Supporting Information), GGH domain 2 contained 15 predicted HuR binding sites, indicating that GGH bound to HuR at multiple sites.

To investigate their respective effects on each other, we knocked down GGH and HuR, respectively. Results revealed that after GGH and HuR knockdown, their respective mRNA expressions were downregulated, yet their protein expressions remained unaffected (Figure S3M–Q, Supporting Information). GGH mRNA expression was positively correlated with HuR in LUAD tissues (Figure S3R, Supporting Information). HuR was reported to be involved in some key biological processes, including DNA replication, cell‐cycle progression, immune function, apoptosis, and oncogenic activities by stabilizing target mRNAs.^[^ ^27^ ^]^ Upon HuR knockdown, KEGG pathway analysis of RNA‐seq data revealed significant enrichment of downregulated genes in cell cycle‐related pathways (Figure S3S, Supporting Information). To identify GGH‐ and HuR‐coregulated genes, we performed RNA‐seq in A549 and H1975 cells, revealing 301 downregulated overlap genes enriched in cell cycle/DNA replication pathways (Figure S3T–V, Supporting Information). The observed changes in mRNA and protein expression of some important regulatory genes, including CCND1 and CDC6, upon HuR knockdown were consistent with those observed upon GGH knockdown (Figure S3W,X, Supporting Information). We speculated that the binding of HuR to GGH was related to their similar regulatory capabilities in modulating the cell cycle and DNA replication pathways.

### 2.4 GGH Binds to mRNAs of CDC6 and CCND1 and Maintains mRNA Stability

**Figure 4:**
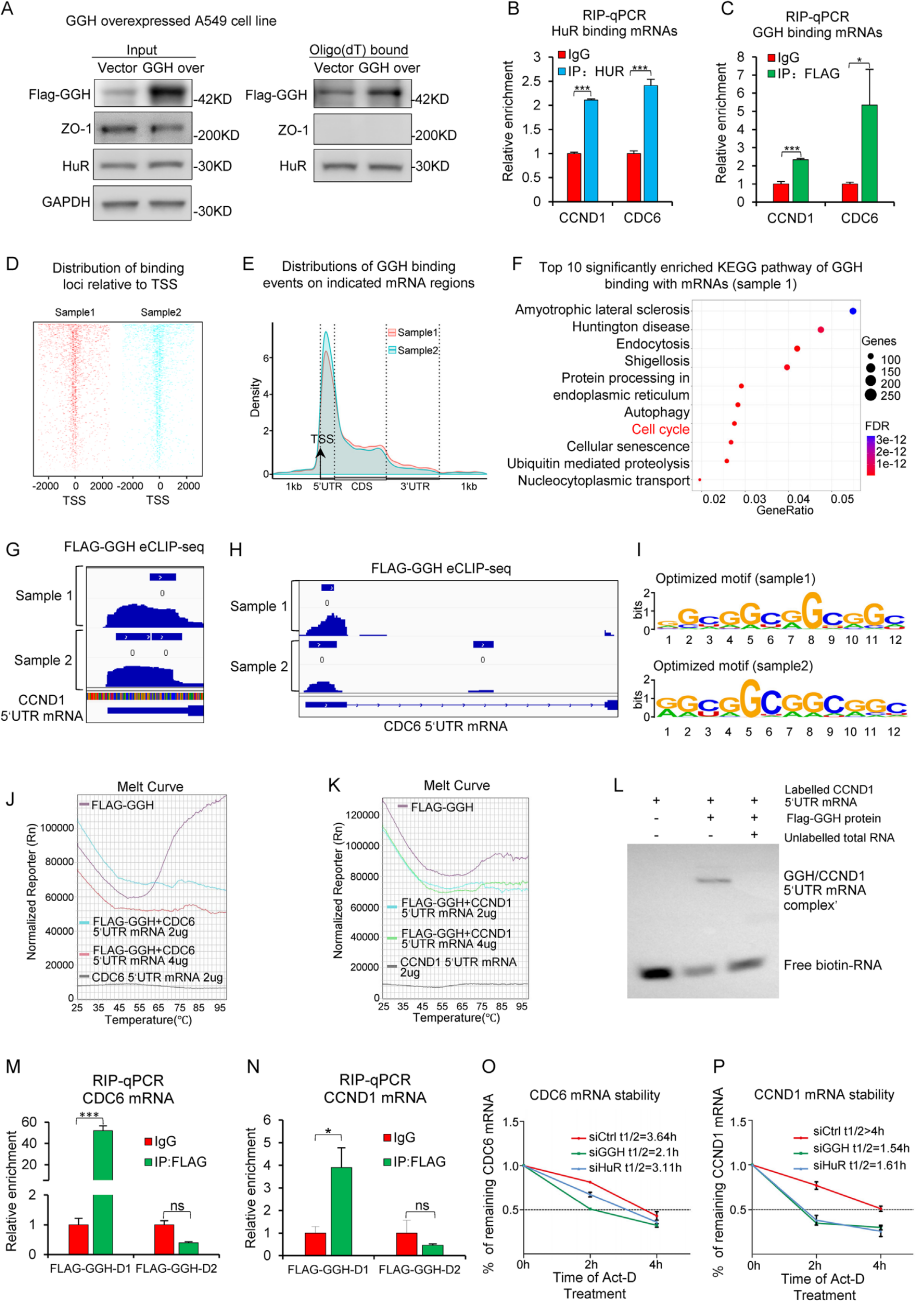
GGH binds to RNA directly. A) Representative western blots for GGH bound to Oligo(dT) beads in A549. HuR is a positive control, and membrane protein ZO‐1 is a negative control for RNA‐binding. Loading control is GAPDH. B) The RIP experiment tests the binding effect of HuR with CDC6 and CCND1 mRNA. C) The RIP experiment tests the binding effect of FLAG‐GGH with CDC6 and CCND1 mRNA. D) Distribution of GGH binding loci relative to transcription start sites (TSS). The left side indicates sample 1 (red) and the right side sample 2 (blue). E) Distributions of GGH binding events on mRNA regions. The red area indicates sample 1 and blue sample 2. F) Enriched KEGG pathways of GGH binding to mRNAs of sample 1. G,H) Integrative Genomics Viewer (IGV) view of the two replicates of GGH eCLIP‐seq showing GGH binding to CCND1 and CDC6 5’UTR mRNAs. I) Sequence logo of the 12bp consensus motif from samples 1 and 2. Each letter's height represents the base's frequency at the corresponding position. *p‐*value of sample1 = 1e‐113, *p‐*value of sample2 = 1e‐154. J,K) Thermal shift assay employs FLAG‐GGH and in vitro transcribed 5’UTR fragments of CDC6 and CCND1 mRNA at different concentrations. Protein melting temperature quantified by RT‐PCR. L) Electrophoretic mobility shift assay (EMSA) of the binding of GGH to the CCND1 5'UTR mRNA in vitro. A biotin‐labeled probe (CCND1 5'UTR mRNA) is used in the EMSA experiment, and a non‐labeled fragment (X100) is used as a competitor. M) The RIP experiment tests the binding effect of FLAG‐GGH‐D1 and FLAG‐GGH‐D2 with CDC6 mRNA. N) The RIP experiment tests the binding effect of FLAG‐GGH‐D1 and FLAG‐GGH‐D2 with CCND1 mRNA. O,P) The stability of CCND1 and CDC6 mRNAs in A549 cells, either depleted of GGH (siGGH) or HuR (siHuR), or not depleted (siCtrl), is determined through ActD pulse‐chase experiments. Cells are exposed to Actinomycin D (ActD) for durations of 0, 2, or 4 hours, and the extracted total RNA is subsequently utilized for qRT‐PCR analysis. The expression levels of the mRNA at each time point are normalized to those of GAPDH mRNA, and the results are plotted as a percentage relative to the initial abundance of each mRNA at the 0‐h ActD treatment time point (set as 100%). The data presented in the figure represent the mean ± SD of three independent experiments. Statistical analysis in panels B, C, M, N was performed using unpaired two‐tailed Student's t‐test, whereas in panels O, P was performed using one‐way ANOVA with multiple comparisons.

To investigate whether GGH functions as an RNA binding protein (RBP), we employed Oligo(dT) beads to pull down polyA mRNAs and assessed whether GGH bound to these mRNAs in A549 GGH overexpression cell line. The results showed that GGH was bound to mRNAs (**Figure** **4**A). To further investigate whether GGH bound to RNA, we used an RNA immunoprecipitation (RIP) experiment. Our data showed that, just like HuR, GGH also bound to the mRNAs of CDC6 and CCND1 (Figure 4B,C). In addition, we performed enhanced crosslinking‐immunoprecipitation and high‐throughput sequencing (eCLIP‐seq) to explore GGH targets (Figure S4A,B, Supporting Information). Most GGH binding sites resided close to the TSS (Figure 4D). Also, GGH bound to both exonic and intronic sites of RNAs, and preferentially in 5’UTRs (Figure 4E). GGH‐bound mRNAs were enriched for diverse diseases, protein transport, maturation, degradation and pathways crucial for cancer progression, including autophagy, cell cycle, and cellular senescence (Figure 4F; Figure S4C, Supporting Information). Of those, GGH indeed bound to cell‐cycle‐related genes CCND1 and CDC6 at 5’UTR regions (Figure 4G,H). Importantly, we identified a GC‐rich consensus motif that was enriched in GGH CLIP peaks, which was primarily detected in the 5’UTR regions of mRNAs (Figure 4I). This motif sequence was observed in several GGH‐bound mRNAs, including multiple genes related to cell cycle and DNA replication (Figure S4D, Supporting Information). We further confirmed that GGH was an RBP by the direct interaction of GGH with CDC6 and CCND1 mRNA in vitro. To do this interaction, we assessed the thermal stability of purified FLAG‐GGH protein with/without the 5’UTR mRNA of CDC6 or CCND1 (Figure 4J,K). The results revealed that the melting temperature of GGH protein was elevated dramatically when co‐incubated with the 5’UTR mRNA of CDC6 or CCND1 in a dose‐dependent manner (Figure 4J,K). What's more, we performed RNA electrophoretic mobility shift assay (RNA EMSA), and found that GGH directly bound to the 5’UTR mRNA of CCND1 (Figure 4L). To further investigate which domain of GGH was responsible for the binding with mRNA, we conducted RIP assays to assess the RNA‐binding capacity of its two domains. The results revealed that GGH domain 1 was bound to the mRNA of CCND1 and CDC6 (Figure 4M,N). These results demonstrated that GGH functions as an RBP, directly bound to 5'UTR mRNAs, with its signal domain responsible for this mRNA interaction.

We next investigated the mechanisms through which GGH and HuR regulated these mRNAs. As a first step, we assessed the effect of GGH or HuR depletion on the expression levels of the CCND1 and CDC6 mRNAs. Similarly, to HuR, the depletion of GGH significantly reduced CCND1 and CDC6 mRNA levels in NSCLC cells (Figure 1J–M; Figures S3X and S4E–H, Supporting Information). To find the possible causes of reduced RNA levels, we quantified RNA and protein levels of RNA polymerase II and basal transcription factors. Results showed that no uniform downward trends across these important transcriptional regulators (Figure S4I–L, Supporting Information). Next, we assessed the effect of GGH or HuR knockdown on the steady state levels of the mRNAs of CCND1 and CDC6. In order to determine whether the decrease in GGH‐bound RNA expression levels was due to a decline in their stability, we performed mRNA stability assay using the transcription inhibitor actinomycin D (ActD). Silencing of GGH and HuR both significantly reduced the stability of CCND1 and CDC6 mRNAs (Figure 4O,P). These results indicated that GGH and HuR regulated the stability of mRNA targets in NSCLC cells.

Further, we evaluated the role of CCND1 and CDC6 in regulating cell cycle and DNA replication via flow cytometry. The results indicated that CCND1 and CDC6 knockdown arrested cell cycle at the G1/S phase in NSCLC cells, just like GGH (Figure S4M–T, Supporting Information). In addition, we performed RNA‐seq analysis upon knockdown of CCND1 or CDC6. We found that the DNA replication and cell cycle pathways were also affected by the down regulated genes of siCCND1 or siCDC6, just like siGGH (Figure S4U,V, Supporting Information), suggesting that GGH in regulating of cell cycle and DNA replication may be through modulating CCND1 and CDC6 mRNA stability. Taken together, these data not only confirmed that GGH bound directly to RNAs and stabilized mRNAs, but also suggested that GGH bound to a group of mRNAs that could regulate cancer development via cell cycle and DNA replication pathways.

### 2.5 GGH, HuR, and mRNA form a Ternary Complex and Require Each Other

**Figure 5:**
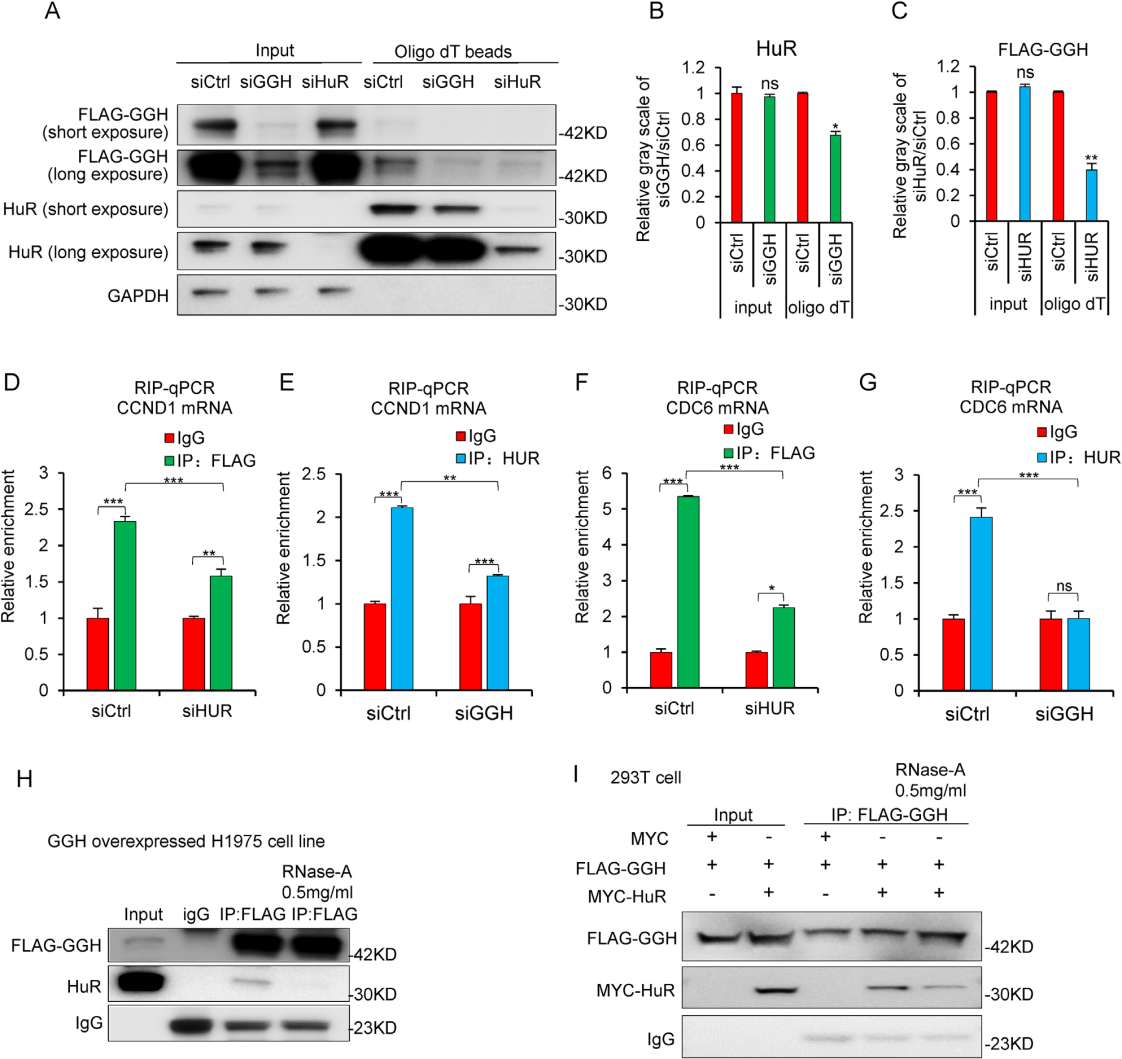
GGH, HuR, and mRNA form a ternary complex and require each other. A) Representative western blots for GGH/HuR bounded to Oligo(dT) beads in A549, after knocking down either GGH or HuR. B) Relative gray scale of HuR was calculated based on Image J densitometry analysis of bands in Figure A. C) Relative gray scale of FLAG‐GGH was calculated based on ImageJ densitometry analysis of bands in Figure A. (D) The RIP experiment tested the binding effect of FLAG‐GGH with CCND1 mRNA after HuR knockdown. E) The RIP experiment tested the binding effect of HuR with CCND1 mRNA after GGH knockdown. F) The RIP experiment tested the binding effect of FLAG‐GGH with CDC6 mRNA after HuR knockdown. G) The RIP experiment tested the binding effect of HuR with CDC6 mRNA after GGH knockdown. H) Co‐IP assays of FLAG‐GGH with or without RNase‐A treatment in H1975 cell line. Immunoglobulin G (IgG) was used as a negative control. I) Co‐IP assays were performed with anti‐FLAG antibody with or without RNase‐A treatment in 293T cell line. Immunoglobulin G (IgG) was used as a negative control. Values were mean ± SD from n = 3 independent experiments. ^*^, *p*<0.05; ^**^, *p* <0.01; ^***^, *p* < 0.001. Statistical analysis in panels B‐G was performed using unpaired two‐tailed Student's t‐test.

To further delineate the mechanism of GGH‐HuR‐mRNA ternary complex, we set out to determine the role of the interaction between HuR and GGH in their binding to RNA. After knocking down either GGH or HuR, we observed a decrease in the remaining protein's RNA binding capacity using an Oligo(dT) bead pulldown assay (**Figure** **5**A–C). Additionally, we conducted RIP experiments to assess the specific RNA‐binding capacity of HuR and GGH following their silencing. Results indicated the interaction between HuR and GGH was required for their binding to the mRNA of CCND1 or CDC6 (Figure 5D–G).

Finally, we also investigated whether RNA was indispensable for GGH binding to HuR. A Co‐IP experiment demonstrated a reduced pull‐down of HuR by FLAG‐GGH after RNase A intervention in GGH overexpression H1975 and 293T cells. The results indicated that the binding capacity between GGH and HuR was reduced after RNA degradation (Figure 5H,I). In addition, Co‐IP experiments showed that the interaction between HuR and either GGH domain 1 or domain 2 requires mRNA. (Figure S5A,B, Supporting Information). These suggested that each component of the GGH‐HuR‐mRNA ternary complex potentially enhanced the binding interactions of the other two.

### 2.6 GGH Silencing or its Inhibitor Azaserine Inhibits Tumor Cell Growth In Vitro and In Vivo

**Figure 6:**
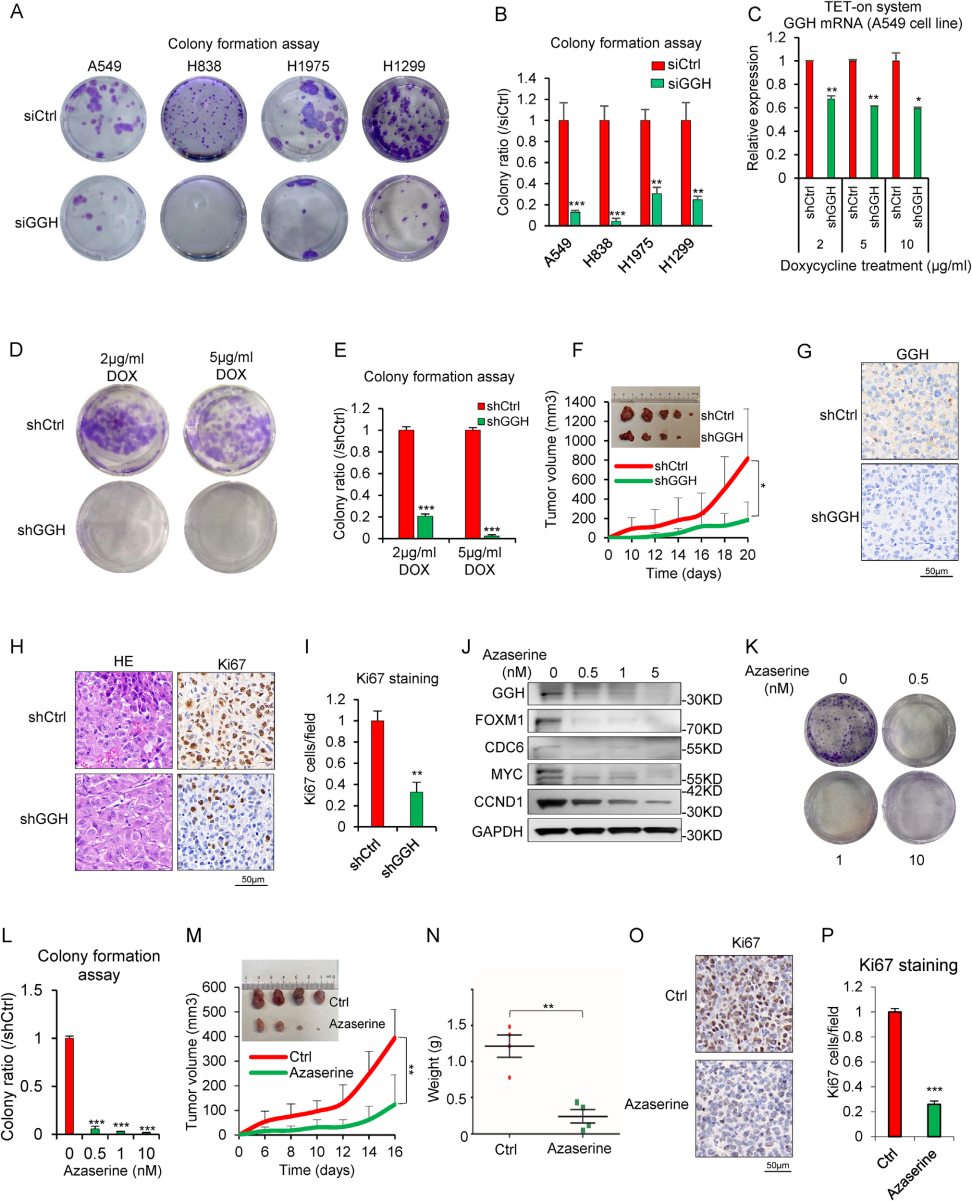
GGH silencing or its inhibiter azaserine inhibits tumor cell growth in vitro and in vivo. A) Colony‐forming ability of before and after siGGH intervention in LUAD cell lines. B) Colony formation ratio (/siCtrl) of each group was calculated in Figure A. ^*^ *p*< 0.05, ^**^ *p*< 0.01, ^***^ *p*< 0.001. C) GGH knockdown efficiency. Tetracycline (Tet) inducible stable A549 cell lines were used in which shRNAs either not targeting any endogenous transcript (Tet‐on‐shCtrl) or targeting GGH (Tet‐on‐shGGH) were expressed under different concentrations of doxycycline (Dox) induction. The knockdown efficiency of GGH was tested by qRT‐PCR. D) Tet‐on‐shGGH inhibited the colony formation abilities of A549 cells in the presence of different Dox concentrations (2, 5µg mL^−1^). E) The statistical chart of the colony formation ability of cells. ^*^ *p* < 0.001, versus Tet‐on‐shCtrl. F) Volume of the tumor‐bearing tissues was monitored and calculated. After tumor inoculation in nude mice (n = 5), tumor sizes were measured every other day, with a total feeding period of 20 days. G) Immunohistochemistry was used to detect the expression of GGH in mouse tumors. Anti‐GGH antibody (1:300 dilution), scale bar = 50 µm. H) Tumor sections were stained with HE and Ki67. Anti‐Ki67 antibody (1:500 dilution), scale bar = 50 µm. I) Quantitative analysis of the Ki67 staining images (n = 3). J) Western blot of GGH, DNA replication and cell cycle‐related proteins with or without azaserine. K) The representative colony formation images of A549 cells in the presence of different concentrations of azaserine (0, 0.5, 1, 10 nm). L) The statistical diagram of colony formation assay. M) Volume of the tumor‐bearing tissues was monitored and calculated (n = 4). N) The weights of tumors among Ctrl and azaserine groups were recorded (n=4). O) Representative Ki67 immunohistochemistry of tumor tissue sections. Anti‐Ki67 antibody (1:500 dilution), scale bar = 50 µm. P) Quantitative analysis of Ki67 staining images (n = 3). ^*^ *p*<0.05, ^**^ *p*<0.01, ^***^ *p*< 0.001. Statistical analysis in panels B, C, E, F, I, L‐N and P was performed by unpaired two‐tailed Student's t‐test.

To determine the oncogenic function of GGH in NSCLC, we performed the colony formation assay. We found that silencing of GGH reduced the colony formation ability in NSCLC cells (**Figure** **6**A,B). Next, we established two stable A549 cell lines in which doxycycline (Dox) induced the expression of either a control shRNA (Tet‐on‐shCtrl) or a shRNA targeting GGH (Tet‐on‐shGGH) (Figure 6C). We found that Tet‐on‐shGGH can inhibit the colony formation abilities of A549 cells in the presence of Dox (Figure 6D,E). We then established a subcutaneous tumor‐bearing mouse model with the control group and experimental group injected respectively using stably transfected TET‐on‐shCtrl and TET‐on‐shGGH A549 cells. Mice were fed a diet containing DOX (200 mg kg^−1^). Mice xenografted with TET‐on‐shGGH A549 cells exhibited a significant decrease in tumor growth compared to those xenografted with TET‐on‐shCtrl cells (Figure 6F). We performed IHC staining of the xenografted tumor tissues for GGH, HE, and a marker of proliferation (Ki67). The results showed that shGGH effectively knocked down GGH expression and inhibited the proliferation of tumor cells in vivo (Figure 6G–I).

Next, we searched for specific inhibitors of GGH. Azaserine, the glutamine antagonist, was reported to act as a GGH inhibitor.^[^ ^28^ ^]^ Western blot experiments demonstrated that azaserine effectively reduced GGH protein expression levels (Figure 6J). Additionally, azaserine inhibited the expression of cell cycle‐related proteins, such as FOXM1, CDC6, RB, and c‐MYC, with trends similar to those observed by GGH knockdown (Figure 6J). We found azaserine inhibited the colony formation abilities of A549 cells (Figure 6K,L). Next, we conducted in vivo experiments using azaserine in mice. Lewis lung carcinoma (LLC) tumors were established in the armpit of immune‐competent C57 mice, and azaserine was intraperitoneally injected twice a week (10 mg kg^−1^). According to the records of tumor volume and tumor weight, azaserine markedly inhibited the growth of subcutaneous tumors of LLC cells (Figure 6M,N). Then, the activity of tumor proliferation was estimated by Ki67 stain. The results showed azaserine inhibited the proliferation of tumor cells in vivo (Figure 6O,P). These results indicated that GGH knockdown or inhibition has anti‐tumor effects in vivo.

### 2.7 GGH is Highly Expressed in Human Lung Cancer Tissues and Higher Expression is Correlated to Poor Patients’ Survival in LUAD

**Figure 7:**
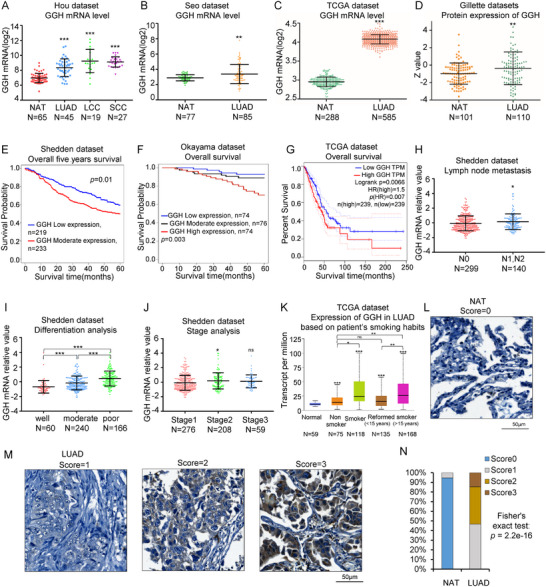
GGH is highly expressed in human lung cancer tissues and higher expression is correlated to poor patient survival in LUAD A) In Hou's data, the mRNA expression of GGH was significantly elevated in diverse NSCLC samples compared to normal lung tissues. NAT: normal lung tissue, LUAD: lung adenocarcinoma, LCC: lung large cell carcinoma, SCC: lung squamous cell carcinoma. B) In Seo's data, the mRNA expression of GGH was significantly elevated in LUAD samples compared to normal lung tissues. C) In TCGA data, the mRNA expression of GGH was significantly elevated in LUAD compared to normal lung tissue. D) In Gillette's data, the protein expression level of GGH was significantly increased in LUAD compared to normal lung tissue. E) In Shedden's data, Kaplan–Meier survival curves showed that high GGH mRNA expression was correlated to poor patient survival in 452 LUAD patients. F) In Okayama's data, Kaplan–Meier survival curves showed that high GGH mRNA expression was associated with poor prognosis of 224 LUAD cases. G) In TCGA data, Kaplan–Meier survival curves showed that high GGH mRNA expression was associated with poor prognosis in 478 LUAD patients. H) GGH was highly expressed in NSCLC patients with lymph node metastasis. N classification (N0: no lymph node metastasis; N1: 1–3 lymph node metastasis; N2: 4–9 lymph node metastasis; N3: ≥ 10 lymph node metastasis). I) GGH mRNA level was increased in poor differentiated LUAD. J) Relationship between the mRNA expression of GGH and the individual cancer stage in LUAD patients. K) Expression of GGH mRNA in LUAD based on patient's smoking habits. L,M) Representative GGH IHC images of NAT and LUAD. GGH intensity in tissue samples was scored in a blinded manner on a scale of 0‐3. IHC 0: No staining, IHC 1: low staining, IHC 2: moderate staining, IHC 3: high staining. NSCLC tissue microarray (TMA) contained 75 individual tumor samples and 75 normal adjacent tissue (NAT) samples. Anti‐GGH antibody (1:200 dilution), scale bar = 50 µm. N) Immunohistochemical quantification was calculated in Figure M. ^*^ *p*< 0.05, ^**^ *p*<0.01, ^***^ *p*< 0.001. Statistical analyses were performed as follows: panels A and I–K used one‐way ANOVA with multiple comparisons; panels B–D and H used unpaired two‐tailed Student's t‐test; panels E–G used Kaplan‐Meier analysis with Log‐rank tests; and panel N used Fisher's exact test.

GGH was reported to play important roles in different cancer types, including NSCLC.^[^ ^29^ ^]^ To explore the expression status of GGH in human lung cancer tissues, we analyzed GGH mRNA expression levels from different databases and found that it was significantly higher in NSCLC (**Figure** **7**A–C).^[^ ^22^, ^30^ ^]^ Similarly, GGH protein expression level was significantly elevated in LUAD samples (Figure 7D).^[^ ^31^ ^]^ The higher expression of GGH was correlated with poor overall patient survival from multiple lung cancer data sets (Figure 7E–G).^[^ ^32^ ^]^ In addition, GGH was highly expressed in LUAD patients with lymph node metastasis, poor differentiation, advanced stages, and a history of long‐term smoking (Figure 7H–K).^[^ ^30^ ^]^ These public datasets proved GGH has an essential oncogenic role in NSCLC. To comprehensively evaluate the diagnostic potential of GGH in LUAD, we performed ROC analyses across three independent cohorts (Hou, TCGA, and Gillette).^[^ ^29^, ^31^ ^]^ Univariate ROC curves demonstrated significant discriminative power of GGH mRNA and protein expression alone (Figure S6A–C, Supporting Information). Critically, we further conducted multiparametric ROC analyses using multivariable logistic regression models incorporating shared clinical covariates. Multivariable ROC models incorporating clinical covariates consistently outperformed the univariate GGH model, as reflected by higher AUC values in each independent cohort (Figure S6D–F, Supporting Information). The incremental AUC confirms that GGH provides complementary diagnostic value beyond established demographic factors, underscoring its potential as a standalone biomarker in clinical decision systems.

To validate the expression of GGH in LUAD, we used immunohistochemistry (IHC) on a tissue microarray to analyze tumor cell‐specific GGH protein levels across a panel of 74 patient‐derived LUAD samples and the paired adjacent normal tissues. Immunohistochemical staining on tissue microarray showed that GGH was significantly elevated in LUAD tissues (*p*< 0.001) (Figure 7L–N). In addition, GGH mRNA expressions were also highly expressed in different cancer types (Figure S6G, Supporting Information). These results illustrated that GGH may serve as an important biomarker in human NSCLC.

## 3 Discussion

RBPs play a crucial role in regulating multiple aspects of cellular RNA, encompassing its synthesis, alternative splicing, polyadenylation, maturation, localization, translation, and degradation.^[^ ^33^ ^]^ In recent years, with the deep understanding of the role of RBPs in cancer biology, researchers have found a close link between these proteins and cancer‐related genes in biological processes.^[^ ^34^ ^]^ RBPs significantly impact a variety of cancer‐related cellular phenotypes, including proliferation, apoptosis, senescence, migration, invasion, and angiogenesis.^[^ ^35^ ^]^ These influences contribute to tumor initiation and progression, as well as affect clinical prognosis. The current state of RNA‐binding proteins shows that they are not complete, but they show great potential in cancer research.

**Figure 8:**
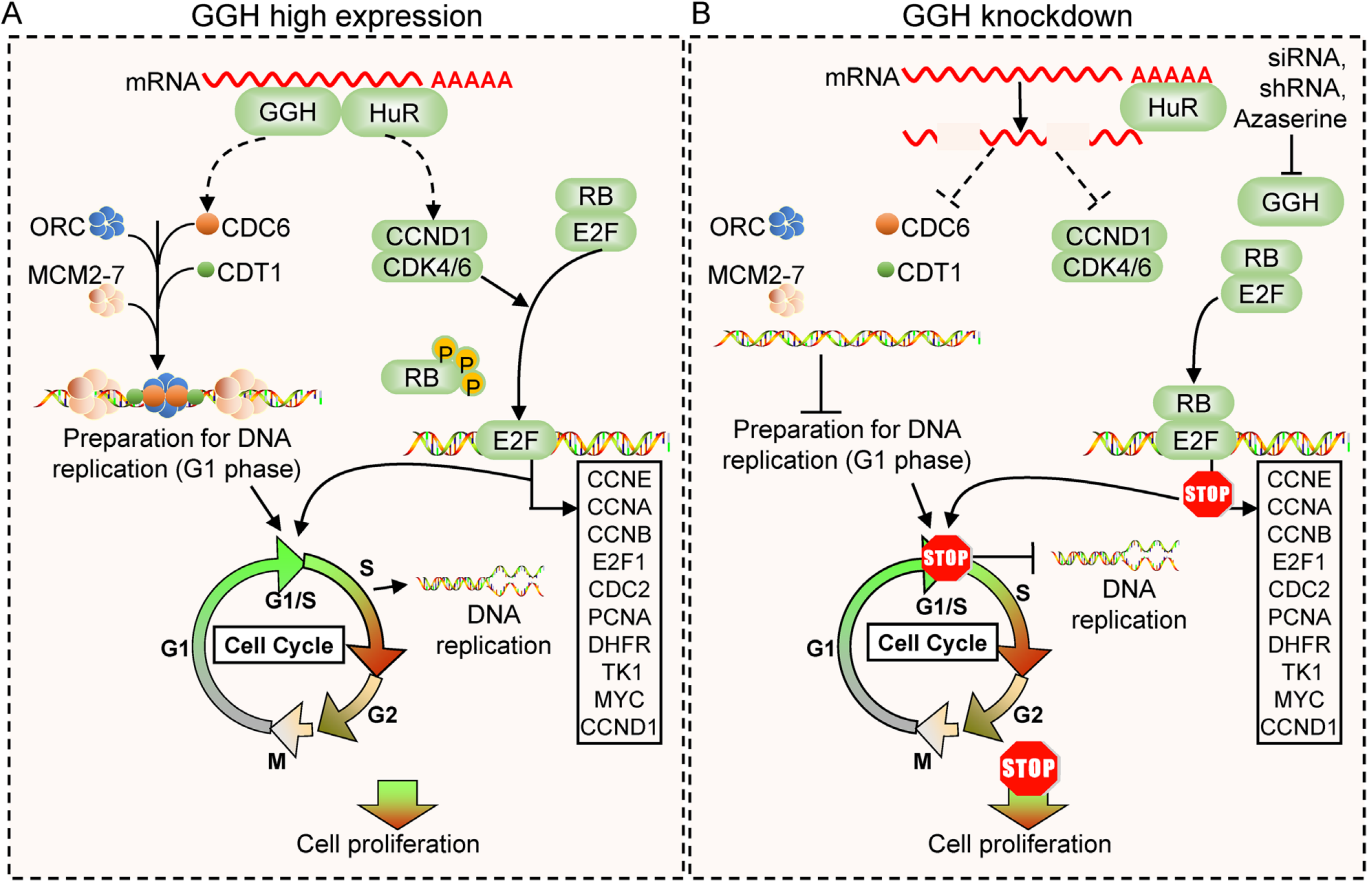
A molecular model depicting the role of the GGH‐HuR complex in stabilizing mRNA to support cell cycle progression and DNA replication. A) When GGH was highly expressed in NSCLC, the GGH‐HuR complex bound to specific mRNAs, particularly those of CDC6 and CCND1, stabilizing these key regulatory transcripts. During the G1 phase, CDC6 functioned as a critical component of the pre‐replication complex, preparing the cell for S‐phase entry and DNA replication. Concurrently, the CCND1‐CDK4/6 complex phosphorylated RB, releasing E2F1‐mediated transcription and facilitating S‐phase entry. Under conditions of high GGH expression, NSCLC cells progressed through the G1/S checkpoint, enabling continuous DNA replication and unrestricted proliferation. B) Conversely, GGH knockdown or azaserine treatment reduced the complex's ability to stabilize mRNAs, resulting in the degradation of CDC6 and CCND1 mRNAs. Consequently, cells were unable to progress through the G1/S checkpoint, DNA replication was impaired, and cell proliferation of NSCLC was suppressed.

In this study, we identified GGH as a novel RBP that stabilizes RNA by binding to high‐GC regions within the 5′ UTR of mRNAs, with its signal domain contributing to this interaction. Using unbiased genome‐wide CLIP‐seq screening, we identified an oncogenic program of hundreds of mRNAs bound by GGH, including those involved in autophagy, cell cycle, and cellular senescence. Combining RNA‐seq data, we found that GGH played a critical role at the post‐transcriptional regulatory level in the cell cycle and DNA replication. It maintained the mRNA stability of some key genes and regulated their expression levels, such as CCND1 and CDC6 (**Figure** **8**A,B).

DNA replication is closely linked with cell cycle regulation, occurring in the S phase and dependent on successful passage through the G1/S checkpoint. In early G1, the assembly of pre‐replication complexes on chromatin is essential for initiating DNA replication and entry into S phase.^[^ ^36^ ^]^ Thus, DNA replication and the cell cycle mutually regulate each other, a process crucial for tumor proliferation. The phosphorylation status of RB in G1 controls cell cycle progression, where phosphorylation by the CCND1‐CDK4/6 complex inactivates RB and releases bound transcription factors, particularly the E2F family. This release activates E2F1, leading to the expression of genes such as DNA polymerase‐α, CDC6, PCNA, DHFR, MYC, TK, CCNE1, and CCND1.^[^ ^4^, ^37^ ^]^ These genes are essential for both the G1/S transition and successful DNA replication during S phase. In eukaryotes, DNA replication initiation involves “initiation licensing,” a sequential recruitment of key protein complexes. The origin recognition complex (ORC) works with CDC6 and CDT1 to load the MCM2‐7 helicase onto replication origins, enabling the start of DNA replication.^[^ ^38^ ^]^ Therefore, GGH deficiency degraded CCND1 and CDC6 mRNA, key regulators of cell cycle and DNA replication, leading to inhibition of DNA replication and cell cycle arrest in G1/S phase, which was consistent with our experimental results (Figure 8A,B).

HuR is a widely studied RNA‐binding protein that stabilizes target RNAs by binding to AU‐rich elements in their 3' UTR.^[^ ^39^ ^]^ It also promotes oncogenesis by stabilizing RNAs crucial for cell cycle and DNA replication, including CDC6, CCND1, MYC, FOS, and p21.^[^ ^16^ ^]^ Our experiment findings confirmed that HuR was capable of binding to and stabilizing the mRNA of CCND1 and CDC6, thereby modulating their expression levels. Moreover, HuR's role in post‐transcriptional regulation extends to its interaction with other RNA‐binding proteins, such as RNPC1, which modulates HuR's activity in regulating p21 mRNA stability.^[^ ^40^ ^]^ In addition, HuR and YB1, both RNA‐binding proteins, interact to stabilize mRNAs that encode proteins involved in muscle cell differentiation and development.^[^ ^41^ ^]^ Our study results revealed that GGH can also bind with HuR to form a trimeric complex with RNA and stabilize the mRNAs of CCND1 and CDC6 (Figure 8A,B). This interaction played a critical role in ensuring smooth cell cycle progression and DNA replication. Notably, both GGH and HuR can independently bind and stabilize RNA. We found that they enhanced each other's binding affinity by binding together, resulting in a more robust stabilization effect on RNA targets. Furthermore, the binding between GGH and HuR required the combined involvement of GGH Domains 1 and 2. Interestingly, while this interaction stabilized target RNAs, it did not affect the expression levels of GGH or HuR themselves. Unlike RNPC1 and YBX, which both bind to the ARE region in the 3′ UTR of mRNA and share binding sites with HuR, GGH bound to the 5′ UTR. Upon forming a complex with HuR, GGH may induce a circular mRNA structure, potentially enhancing RNA stability. This interplay highlighted the complexity of GGH's regulatory network, as it not only stabilized mRNAs but also collaborated with other factors to fine‐tune gene expression in response to cellular signals.

This study has discovered that the non‐canonical function of GGH was capable of maintaining cell cycle progression and DNA replication, which was in contrast to its classical enzymatic role. On the one hand, the folate hydrolase GGH lowers intracellular polyglutamylated folate levels by hydrolyzing polyglutamate forms of folate.^[^ ^42^ ^]^ This reduction in polyglutamate levels subsequently downregulates the purine supply, thereby inhibiting DNA synthesis within the cells. On the other hand, as an RNA‐binding protein, GGH collaborated with HuR to stabilize the mRNAs of key regulatory factors involved in the cell cycle and DNA replication. This interaction was crucial for maintaining the proper progression of DNA replication and ensuring the smooth operation of the cell cycle. The mechanism of GGH to balance these conflicting roles was unknown. However, this regulatory balance was crucial for maintaining cellular homeostasis. Our findings indicated that GGH functions more effectively as an RNA‐binding protein in tumor cells, promoting the smooth progression of the cell cycle and DNA replication. Evidence for this came from comparing downregulation pathways following GGH knockdown in NSCLC cell lines and lung epithelial cells. In NSCLC cells, the cell cycle and DNA replication pathways were significantly downregulated, whereas in epithelial cells, these pathways showed only minor downregulation. Compared to lung epithelial cells, silencing of GGH also had a stronger ability in G1/S phase arresting in NSCLC cells. Additionally, in human LUAD tissues, GGH expression correlated genes were strongly involved in cell cycle and DNA replication pathways, while it was weaker in adjacent non‐cancerous tissues. This distinction may be due to cancer cells having rapid cell cycle progression and extensive DNA replication.

We further investigated strategies to impede tumor growth in mice by knocking down GGH expression and utilizing a specific inhibitor, azaserine. Both strategies effectively suppressed tumor growth in vivo, further confirming the critical role of GGH in tumorigenesis. We knew that azaserine might not be an ideal inhibitor of GGH as an RNA‐binding protein, since it is primarily a glutamine inhibitor with multiple functions. However, we found that it could effectively reduce GGH protein levels and subsequently downregulate the expression of genes in the cell cycle and DNA replication pathways. Future study may focus on identifying the specific binding sites of GGH signal domain with RNA, potentially leading to the discovery of small molecule targeted drugs that can effectively inhibit GGH's RNA‐binding activity, thereby achieving the goal of tumor growth suppression.

In summary, GGH has an oncogenic role in lung cancer progression, which may act as an RNA‐binding protein together with HuR protein in the regulation of cell cycle and DNA replication processes. Future studies may focus on understanding the relationship between GGH and cancer progression from the perspective of RNA centers, which may provide new mechanisms and methods for cancer diagnosis and treatment.

## 4 Experimental Section

### Cell Lines

Human NSCLC cell lines A549, H1975, H1299, and H838 were purchased from Cellcook Biotech (Guangzhou, China). Lung epithelial cell line BEAS‐2B and human embryonic kidney 293T cells were kindly gifted by Prof. Jian Zhang (Southern University of Science and Technology). All cell lines were confirmed by short‐tandem repeat (STR) analysis, and no mycoplasma contamination was certified by using a Mycoplasma Detection Kit (TransGen Biotech).

### Cell Culture

Cells were cultured in RPMI 1640 and DMEM medium (Gibco) supplemented with 10% FBS (Gibco) in a humidified incubator with 5% CO_2_ at 37 °C.

### siRNA Transfection

Cells were trypsinized and seeded in 6‐well plates. Transfection was performed at 30% confluence. For siRNA‐lipid complexes, 2.5 µL siRNA and 1 µL LipoRNAiMAX were separately diluted in 100 µL Opti‐MEM each, incubated for 5 min, then combined and mixed thoroughly. After 15 min incubation at room temperature, the mixture (200 µL) was added to each well containing 1.8 mL medium. RNA and protein were harvested at 48 and 72 h post‐transfection, respectively. siRNAs demonstrating high knockdown efficiency were pooled for subsequent experiments.

### Cell Proliferation Assay

Cells were seeded into 96‐well plates at optimized densities (A549: 800 cells per well; H1975: 1000 cells per well; H838: 1500 cells per well; H1299: 800 cells per well; six replicates per experimental group). After 24 h of adherence, cells were transfected with siRNAs. CCK‐8 assay was performed at 24‐h intervals (24, 48, 72, 96, and 120 h post‐transfection). Briefly, 10% (v/v) CCK‐8 reagent was added to each well, followed by incubation at 37 °C for 1 h. Absorbance at 450 nm was measured using a Bio‐Rad Microplate Reader, with cell‐free wells serving as blank controls for background subtraction.

### Cell Transfection

The cells were digested and plated, and the 6‐well plate was observed until the cell density reached 30%. Dilute LipoRNAiMAX and siRNA with OPTI‐MEM medium and let it sit at room temperature for 5 min. Added LipoRNAiMAX to the diluted siRNA, mixed well, and incubated at room temperature for 15 min. Added 200 µL of the incubated mixture to each well of the 6‐well plate (pre‐filled with 1.8 mL medium). RNA was extracted after 48 h for detection, and protein was extracted after 72 h for detection.

### Colony Formation Assay

Transfected NSCLC cells were digested with trypsin, centrifuged, resuspended, and counted. A suspension containing 800 cancer cells was added to each well of a 6‐well plate, with control and experimental groups prepared in triplicate. The cells were incubated at 37 °C with 5% CO_2_ and 95% humidity for ≈10 days. After incubation, the cells were fixed with methanol and stained with crystal violet. Finally, the number of colonies was counted and analyzed.

### Western Blot

Total protein was extracted from cultured cells using RIPA lysis buffer containing protease/phosphatase inhibitors, followed by concentration measurements with the BCA Protein Assay Kit. To standardize loading, lysates were diluted with RIPA buffer to 1 mg mL^−1^, mixed with 5× SDS‐PAGE loading buffer at a 1:4 ratio (v/v), and denatured at 95 °C for 10 min. A fixed volume of 20 µL (20 µg total protein) was loaded per lane. SDS‐PAGE was performed at 150 V for 50 min, and then the proteins were transferred to a PVDF membrane using a 200 mA constant current for 2 h. The PVDF membrane was blocked in 5% non‐fat milk in TBST for 1 h, and the primary antibody was added, incubating on a shaker at 4 °C overnight. After washing the membrane, the secondary antibody was added, and incubation was carried out at 37 °C for 1 h. After washing, the membrane was exposed using chemiluminescent detection, and GAPDH protein bands were detected as internal controls for each sample.

### qRT‐PCR

Total RNA was extracted according to the kit's instructions (Vazyme). After quantification using NanoDrop 2000, total RNA was reverse transcribed using the HiScript II Q RT SuperMix for qPCR Kit (Vazyme) according to the instructions. The resulting product was analyzed by qRT‐PCR using Taq Pro Universal SYBR qPCR Master Mix (Vazyme).

### Cell Cycle Assay

After 48 h of siRNA transfection, cells were digested, centrifuged, and treated with 70% ethanol, then stored overnight at 4 °C. The next day, the cells were centrifuged and washed twice with PBS and stain buffer. Cells were stained with Propidium Iodide (PI, 1× final concentration diluted from 20× stock) and RNase A (1× final concentration diluted from 50× stock) in the dark at room temperature for 30 min. The samples were protected from light and stored at 4 °C for analysis. Flow cytometry was performed using a BD Biosciences instrument equipped with a 488 nm laser, with 50 000 events acquired per sample. Data were processed using ModFit LT software (v3.1) to quantify cell cycle distribution (G0/G1, S, G2/M phases).

### Co‐IP Assay

Cells were lysed with NP40 lysis buffer (1:50 protease inhibitor, 1:100 RNase inhibitor). After centrifugation, the supernatant was collected and incubated overnight with the target protein antibody or IgG antibody (Cell Signaling Technology) on a rotator. The next day, Dynabeads Protein G (Invitrogen) was added for immunoprecipitation. The immune complexes were collected, and Western blot analysis was performed to detect proteins associated with the target protein.

### Oligo dT Beads Pulldown Assay

Cells were washed twice with ice‐cold PBS, and ice‐cold NP40 buffer (1:50 protease inhibitor, 1:100 RNase inhibitor) was added. The cells were scraped into a 1.5 mL EP tube. Cells were homogenized on ice for 10 min and vortexed 10 times. The lysate was centrifuged at 14,000 ×g at 4 °C, and the supernatant was collected. All samples were normalized to equal protein concentration (measured by Bradford assay) before loading. Specifically, 5% of the total lysate was reserved as Input, while the remainder was added to swollen oligo (dT) beads (Invitrogen). Samples were rotated end‐to‐end at 4 °C for 2 h, followed by washing the beads 5 times with washing buffer. The sample was eluted from the beads using 2× loading buffer containing 10 mm DTT, boiled at 95 °C for 10 min, and vortexed once per minute. The samples were analyzed by Western blot.

### Immunofluorescence

Coverslips in 6‐well plates were cultured for 48 h. Cells were fixed with 4% paraformaldehyde for 15 min at room temperature, followed by permeabilization with 0.5% Triton X‐100 for 20 min. After three PBS washes, non‐specific binding was blocked with 10% goat serum for 30 min. Primary antibodies were applied (Mouse anti‐FLAG M2, 1:100; HuR/ELAVL1 Rabbit mAb, 1:100), and the samples were incubated overnight at 4 °C in a humidified chamber. After three washes with PBST (PBS + 0.05% Tween‐20), fluorescent secondary antibodies were added (AF488‐Goat anti‐Mouse IgG, 1:400; Alexa Fluor 594‐Goat anti‐Rabbit IgG, 1:400), and incubated for 1 h at room temperature protected from light. Nuclei were counterstained with DAPI for 5 min in the dark. After removing excess liquid, coverslips were mounted with Antifade Mounting Medium. Images were captured using a Zeiss LSM 980 confocal microscope with 63× oil objectives. Utilized ImageJ plugins (ScatterJ) to calculate Pearson's Correlation Coefficient (PCC).

### Immunohistochemistry

The TMA (Tissue array) chips (Shanghai Outdo Biotech Co., Ltd.) were stained for immunohistochemistry. After baking at 60 °C for 2–4 h, the sections were dewaxed in xylene and rehydrated in a graded series of ethanol. Antigen retrieval was performed using sodium citrate buffer, and sections were blocked with 3% H_2_O_2_. Primary antibodies were incubated overnight at 4 °C, and secondary antibodies were applied for 30 min. The sections were stained with hematoxylin, mounted, and images were captured. The TMA used in this study was approved by the Ethics Committee of Shanghai Outdo Biotech Company (Project No. HLugA150CS03; Approval No. SHYJS‐CP‐1901005).

### RIP Assay

Cells were plated in 15 cm dishes and treated with siRNA. After 72 h, cells were scraped, centrifuged, and the supernatant was discarded. RNA was extracted from the magnetic beads using the RNA Immunoprecipitation (RIP) kit (GENESEED) and then reverse transcribed for RT‐PCR analysis.

### CLIP‐seq

GGH‐overexpressing H1975 cells were plated in 15 cm dishes. Once fully confluent, the cells were irradiated at 254 nm with 400 mJ cm^−^^2^ using a UVP crosslinker. Cells were then scraped, centrifuged, and the supernatant was discarded. The cell pellet was resuspended in PBS and stored for transport on dry ice to Dacheng Bio (Shanghai) for sequencing.

### Electrophoretic Mobility Shift Assay

The 5' UTR mRNA fragments of CCND1 and CDC6 were transcribed in vitro and labeled with biotin at the 5' end. FLAG‐GGH protein was purified. The experiment was conducted using the Thermo Scientific LightShift Chemiluminescent RNA EMSA Kit. Non‐denaturing polyacrylamide gel electrophoresis (PAGE) was performed, followed by transfer to a nylon membrane. After UV cross‐linking, chemiluminescent detection was carried out.

### Fluorescent Thermal Shift Assay

In vitro transcribed 5' UTR mRNA fragments of CCND1 and CDC6 and purified GGH protein were prepared. Protein samples were mixed with mRNA fragments or RNase‐free water. Protein melting temperature was measured by qPCR, and the thermal stability of GGH was used to determine binding to the mRNA fragments.

### RNA Sequencing

RNA sequencing was performed by Guangzhou Genedenovo Biotechnology Co., Ltd. Briefly, total RNA was extracted using TRIzol reagent 48 h after siRNA knockdown of target genes (GGH, HuR, FPGS, CCND1, and CDC6) in NSCLC and lung epithelial cell lines. RNA concentration, purity, and integrity (RIN > 7) were verified via spectrophotometry (OD260/280) and Agilent Bioanalyzer. Ribosomal RNA‐depleted mRNA was enriched, and sequencing libraries were constructed by Oligo(dT) bead‐based polyA capture, followed by ultrasonication fragmentation, cDNA synthesis, Illumina adapter ligation, size selection (200 ± 50 bp), and Phusion High‐Fidelity PCR amplification. Libraries were sequenced on the Illumina NovaSeq platform (paired‐end 150 bp). Raw reads were quality‐filtered (fastp) to generate clean reads, which were aligned to the Ensembl genome (release 100) using HISAT2 for transcript quantification (RSEM), splice variant detection, and SNP analysis. Sample reproducibility was validated by principal component analysis (PCA) and Pearson correlation. Differentially expressed genes (DEGs, FDR < 0.05) were identified using edgeR with Benjamini–Hochberg correction. Pathway enrichment was analyzed via Gene Set Enrichment Analysis (GSEA) ranked by signal‐to‐noise ratio.

### Proteomic Analysis by Data‐Independent Acquisition (DIA) Mass Spectrometry

Cells were transfected with siRNAs for 72 h, lysed in lysis buffer (1:100 protease inhibitor), and rotated at 4 °C for 30 min. Lysates were centrifuged, and protein concentrations were quantified by the BCA method. Proteins were denatured, reduced, alkylated, and digested with trypsin. Peptides were separated on the UltiMate 3000 HPLC system, and DIA data were acquired in the diaPASEF mode. Raw data were processed using Spectronaut software with default settings. Quantification was normalized, and differential proteins were identified (FDR cutoff on precursor level was 1% and protein level was 1%).

### Animal Experiments

C57 and B/c Nude mice were purchased from Beijing Weitong Lihua Experimental Animal Technology Co., LTD. All animal experimental procedures were ethically approved by the Institutional Animal Care and Use Committee of the Southern University of Science and Technology (Ethics Acceptance Number: SUSTech‐SL2024022713).

The TET‐on system was established in A549 cells, that A549 cells were infected with TetIIP‐Turbo RFP‐MCS (MIR30)‐Ubi‐TetR‐IRES Puromycin lentiviruses, followed by selection with 2 µg mL^−1^ puromycin. The stable A549 cell lines (5  ×  10^6^) were subcutaneously injected into 5–8 week‐old B/c Nude mice. Mice were then fed with a diet containing DOX (200 mg kg^−1^) (Jiangsu Xietong Pharmaceutical Bio‐engineering Co., Ltd.). Tumor sizes were measured every other day, with a total feeding period of 20 days.

We subcutaneously injected Lewis lung carcinoma (LLC) (5X10^6^) into 5–8 week‐old C57 mice. Azaserine (10 mg kg^−1^) and ddH2O were administered via intraperitoneal injection to the Azaserine and Ctrl groups of mice, respectively, at a dose of 100 microliters twice a week. Tumor sizes were measured every other day, with a total feeding period of 16 days. Mice were always euthanized following the painless cervical dislocation. The size of the tumors was measured using a caliper and the volume was calculated as π/6 × width  ×  width  ×  length (mm^3^).

### Public Data Resources

This study integrated multi‐source biomedical databases and clinical datasets to investigate the correlation between gene expression signatures and tumor prognosis. RNA expression profiles (RNA‐seq/microarray), protein expression profiles and clinical parameters (e.g., survival time, TNM stage and differentiation status) were obtained from TCGA‐LUAD cohort (http://cancergenome.nih.gov) via UCSC Xena platform, Hou dataset (GSE19188, 91 NSCLC and 65 adjacent tissues),^[^ ^29^ ^]^ Seo dataset (GSE40419, 77 LUAD and 87 matched normal tissues),^[^ ^22^ ^]^ Shedden dataset (442 LUAD tissues with comprehensive clinical annotations, including TNM stage, differentiation status and survival time),^[^ ^32a^ ^]^ Okayama dataset (GSE31210, 226 stage I–II LUADs)^[^ ^32b^ ^]^ and Gillette dataset (CPTAC data portal, https://cptac‐data‐portal.georgetown.edU/cptac/s/S056, 110 LUAD and 101 normal tissues with proteogenomic profiles).^[^ ^31^ ^]^

Gene‐gene correlations were assessed using Pearson correlation analysis based on integrated RNA expression profiles from the UM (67 LUAD),^[^ ^21^ ^]^ Seo,^[^ ^22^ ^]^ and TCGA‐LUAD datasets (tumor and matched normal tissues).

### Statistical Analysis

Data were analyzed using Microsoft Excel 365, GraphPad Prism 9.0, and R 4.1.3. Pre‐processing data of public datasets was limited to log2 transformation. Normally distributed continuous data were analyzed using unpaired two‐tailed Student's t‐test (for two‐group comparisons) or one‐way ANOVA with multiple comparisons (for multiple groups), and categorical data were analyzed using Fisher's exact test. All experiments included ≥ 3 independent biological replicates. Data are presented as mean ± SD. Statistical significance was defined as *p* < 0.05. For RNA‐seq differential expression analysis and eCLIP‐seq‐based enrichment pathways of GGH‐bound RNAs, significant pathway enrichment (analyzed by DAVID v6.8) was defined as FDR < 0.05.^[^ ^43^ ^]^

## Conflict of Interest

The authors declare no conflict of interest.

## Author Contributions

Y.L. and G.C. conceived and designed the experiments, and wrote the manuscript; Y.L., X.L., Y.D., S.C., X.H., Z.X., Z.Z., H.Z., and X.Z. performed the experiments and analyzed data; G.C. performed conceptualization, supervision, and funding acquisition. All authors read and approved the final manuscript.

## Supporting information



Supporting Information

## Data Availability

The data that support the findings of this study are available from the corresponding author upon reasonable request.
