# Supplementary material for: The GGH/HuR Complex Binds and Stabilizes mRNAs to Maintain Tumor Cell Cycle and DNA Replication
Source: Adv Sci (Weinh). 2025 Aug 19;12(42):e00838. doi: 10.1002/advs.202500838 (PMC12622440; doi:10.1002/advs.202500838)
Supplement: Supplementary file 1 — Supporting Information [file ADVS-12-e00838-s001.pdf]

Figure S1

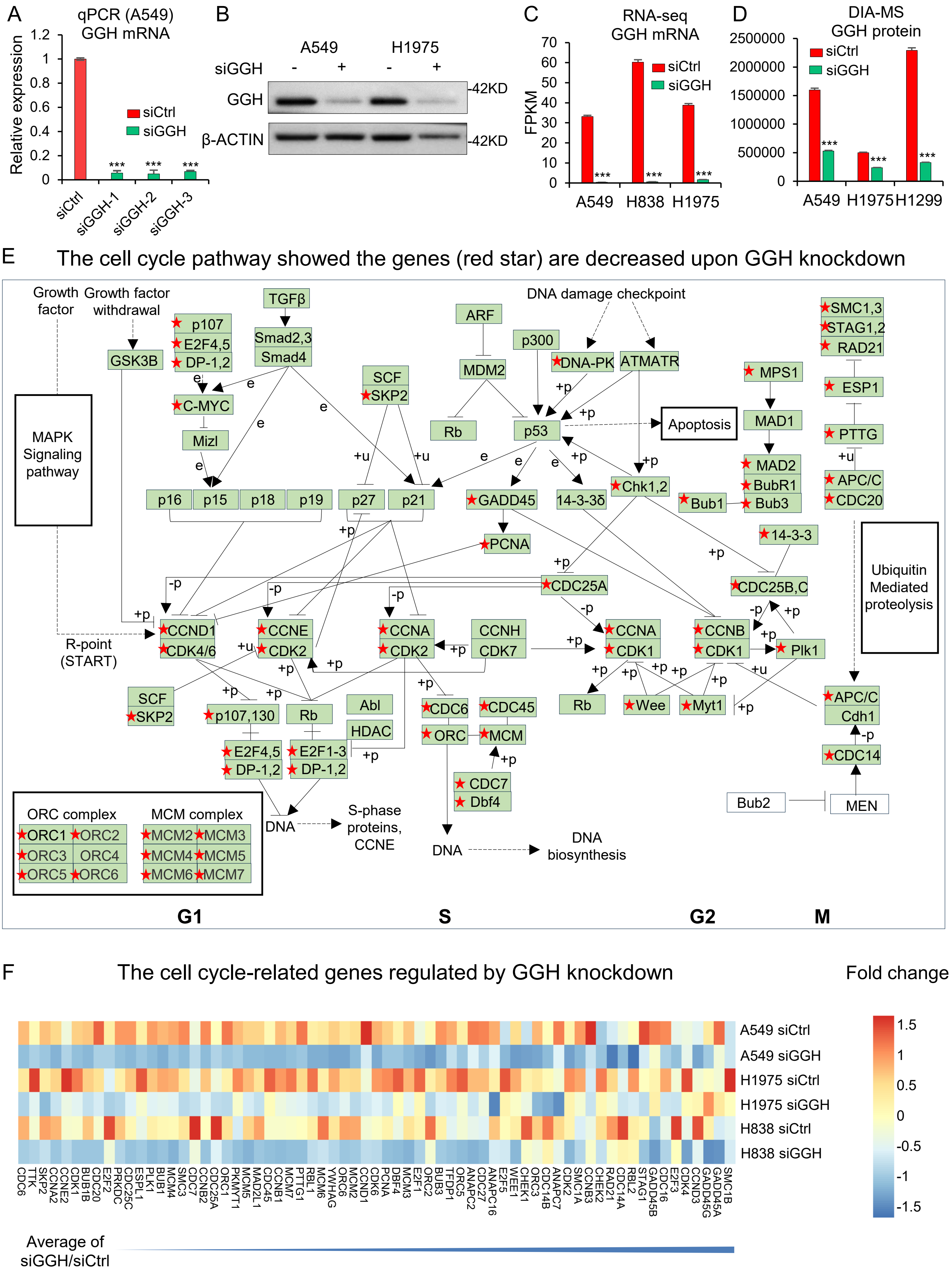
A
qPCR (A549)
GGH mRNA
Relative expression
1.2
1
0.8
0.6
0.4
0.2
0
siCtrl
siGGH
***
siCtrl
siGGH-1
siGGH-2
siGGH-3
B
A549
H1975
siGGH
GGH
β-ACTIN
-42KD
-42KD
C
RNA-seq
GGH mRNA
FPKM
70
60
50
40
30
20
10
0
A549
H838
H1975
D
DIA-MS
GGH protein
2500000
2000000
1500000
1000000
500000
0
A549
H1975
H1299
E
The cell cycle pathway showed the genes (red star) are decreased upon GGH knockdown
Growth factor
Growth factor withdrawal
MAPK Signaling pathway
GSK3B
TGFβ
Smad2,3
Smad4
p107
E2F4,5
DP-1,2
C-MYC
Mizl
p16
p15
p18
p19
SCF
SKP2
p27
p21
ARF
MDM2
Rb
p300
DNA damage checkpoint
DNA-PK
ATMATR
p53
Apoptosis
GADD45
14-3-3δ
Chk1,2
PCNA
CDC25A
MPS1
MAD1
MAD2
BubR1
Bub1
Bub3
14-3-3
CDC25B,C
SMC1,3
STAG1,2
RAD21
ESP1
PTTG
APC/C
CDC20
Ubiquitin Mediated proteolysis
R-point (START)
CCND1
CDK4/6
CCNE
CDK2
CCNA
CDK2
CCNH
CDK7
CCNA
CDK1
CCNB
CDK1
Plk1
p107,130
Rb
Abl
HDAC
E2F4,5
DP-1,2
E2F1-3
DP-1,2
CDC6
ORC
CDC45
MCM
CDC7
Dbf4
Rb
Wee
Myt1
APC/C
Cdh1
CDC14
Bub2
MEN
ORC complex
ORC1
ORC2
ORC3
ORC4
ORC5
ORC6
MCM complex
MCM2
MCM3
MCM4
MCM5
MCM6
MCM7
DNA
S-phase proteins, CCNE
DNA
DNA biosynthesis
G1
S
G2
M
F
The cell cycle-related genes regulated by GGH knockdown
Fold change
A549 siCtrl
A549 siGGH
H1975 siCtrl
H1975 siGGH
H838 siCtrl
H838 siGGH
1.5
1
0.5
0
-0.5
-1
-1.5
CDC6 TTK SKP2 CCNE2 CDK1 BUB1B CDC20 E2F2 PRKDC CDC25C ESPL1 PLK1 BUB1 MCM4 SMC3 CDC7 CCNB2 CDC25A ORC1 PKMYT1 MCM5 MAD2L1 CDC45 CCNB1 MCM7 PTTG1 RBL1 MCM6 YWHAG ORC6 MCM2 CCND1 CDK6 PCNA DBF4 MCM3 E2F1 ORC2 BUB3 TFDP1 ORC5 ANAPC2 CDC27 ANAPC16 E2F5 WEE1 CHEK1 ORC3 CDC14B ANAPC7 CDK2 SMC1A CCNB3 CHEK2 RAD21 CDC14A RBL2 STAG1 GADD45B CDC16 E2F3 CDK4 CCND3 GADD45G GADD45A SMC1B
Average of siGGH/siCtrl

Figure S1

G The DNA replication pathway showed the genes (red star) are decreased upon GGH knockdown

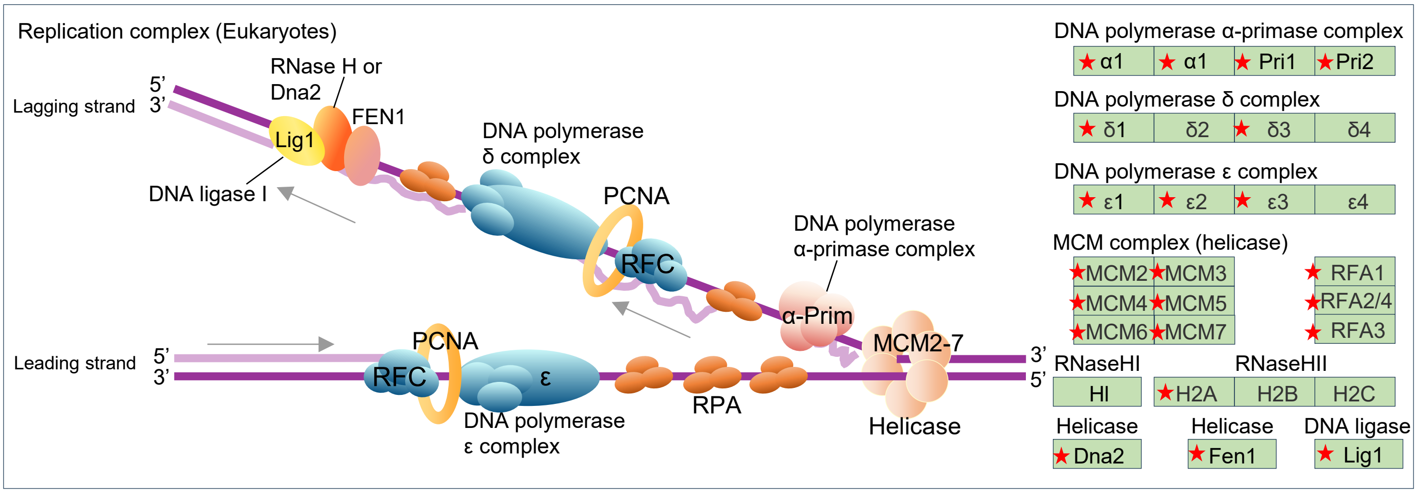

H The DNA replication-related genes regulated by GGH knockdown

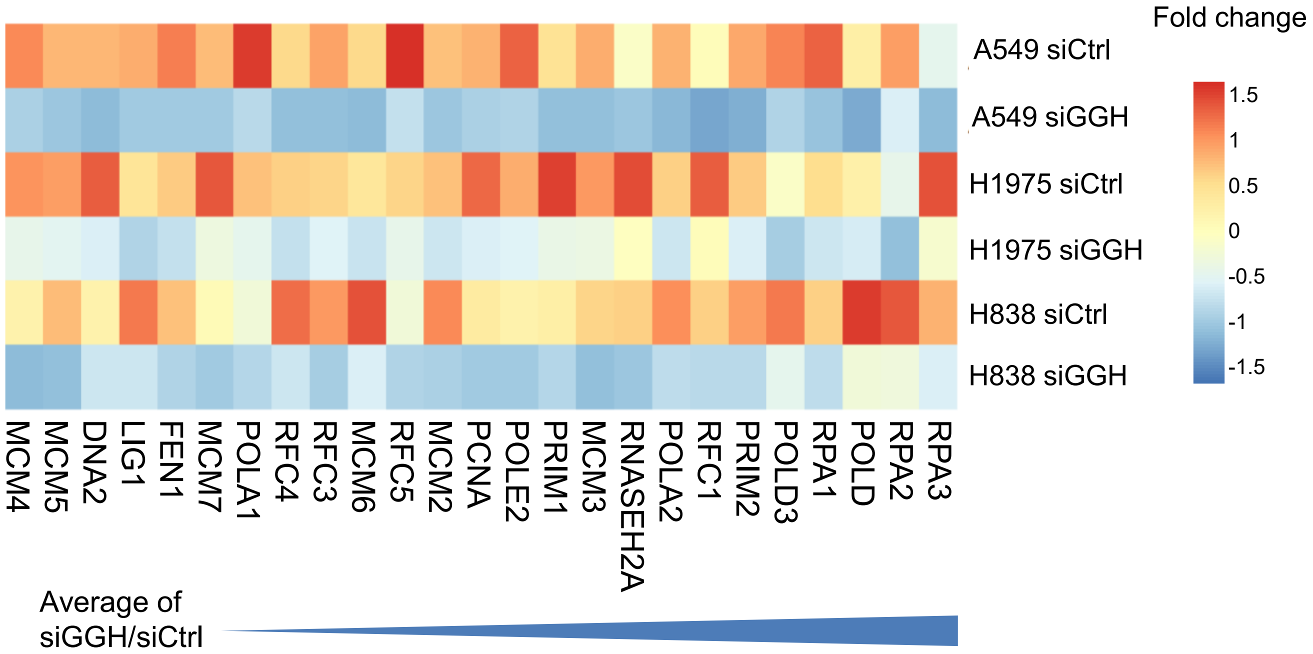

Figure S1

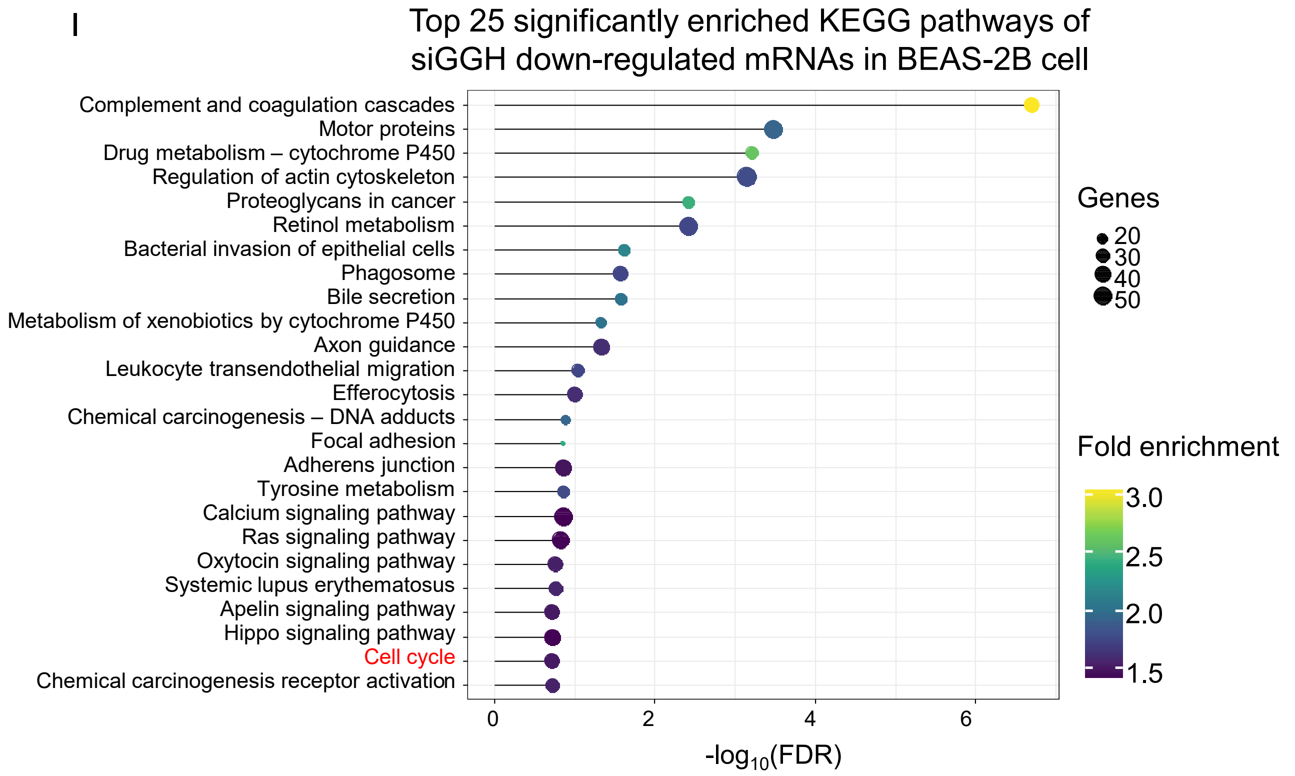
I
Top 25 significantly enriched KEGG pathways of siGGH down-regulated mRNAs in BEAS-2B cell
Complement and coagulation cascades
Motor proteins
Drug metabolism – cytochrome P450
Regulation of actin cytoskeleton
Proteoglycans in cancer
Retinol metabolism
Bacterial invasion of epithelial cells
Phagosome
Bile secretion
Metabolism of xenobiotics by cytochrome P450
Axon guidance
Leukocyte transendothelial migration
Efferocytosis
Chemical carcinogenesis – DNA adducts
Focal adhesion
Adherens junction
Tyrosine metabolism
Calcium signaling pathway
Ras signaling pathway
Oxytocin signaling pathway
Systemic lupus erythematosus
Apelin signaling pathway
Hippo signaling pathway
Cell cycle
Chemical carcinogenesis receptor activation
0
2
4
6
-log10(FDR)
Genes
20
30
40
50
Fold enrichment
3.0
2.5
2.0
1.5

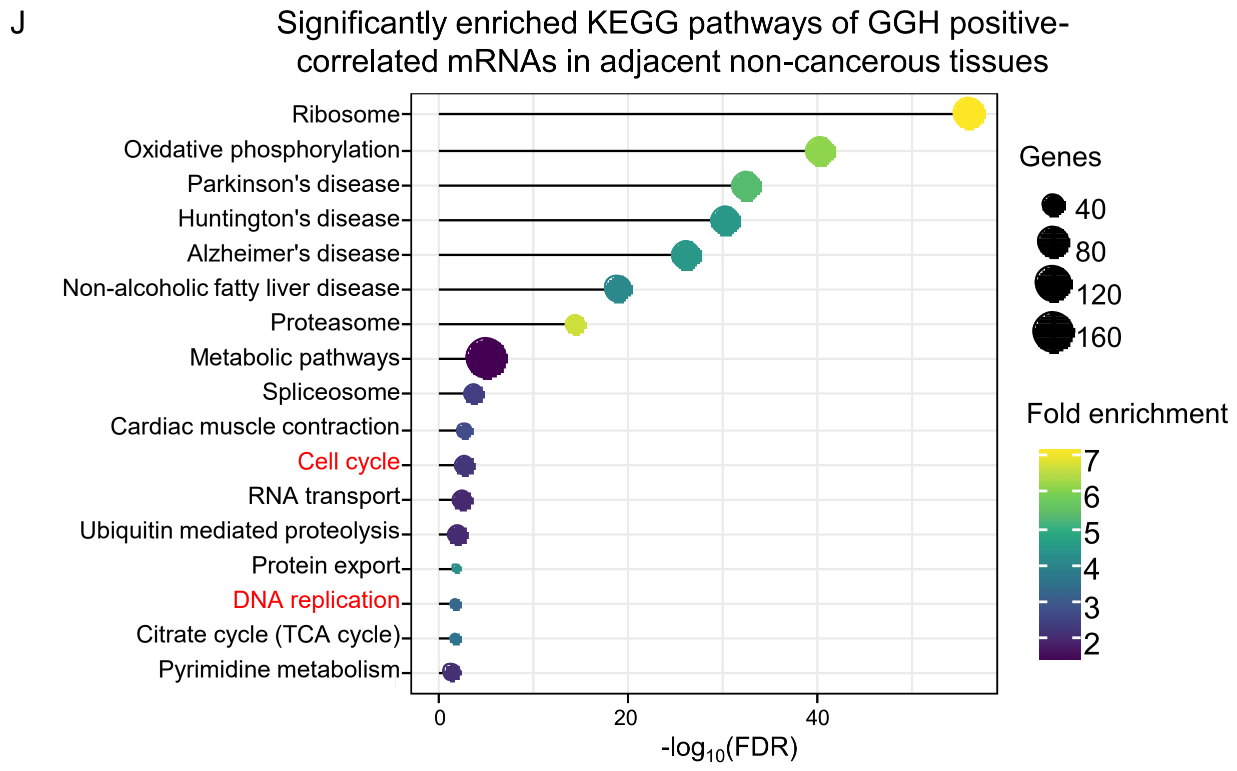
J
Significantly enriched KEGG pathways of GGH positive-correlated mRNAs in adjacent non-cancerous tissues
Ribosome
Oxidative phosphorylation
Parkinson's disease
Huntington's disease
Alzheimer's disease
Non-alcoholic fatty liver disease
Proteasome
Metabolic pathways
Spliceosome
Cardiac muscle contraction
Cell cycle
RNA transport
Ubiquitin mediated proteolysis
Protein export
DNA replication
Citrate cycle (TCA cycle)
Pyrimidine metabolism
0
20
40
-log10(FDR)
Genes
40
80
120
160
Fold enrichment
7
6
5
4
3
2

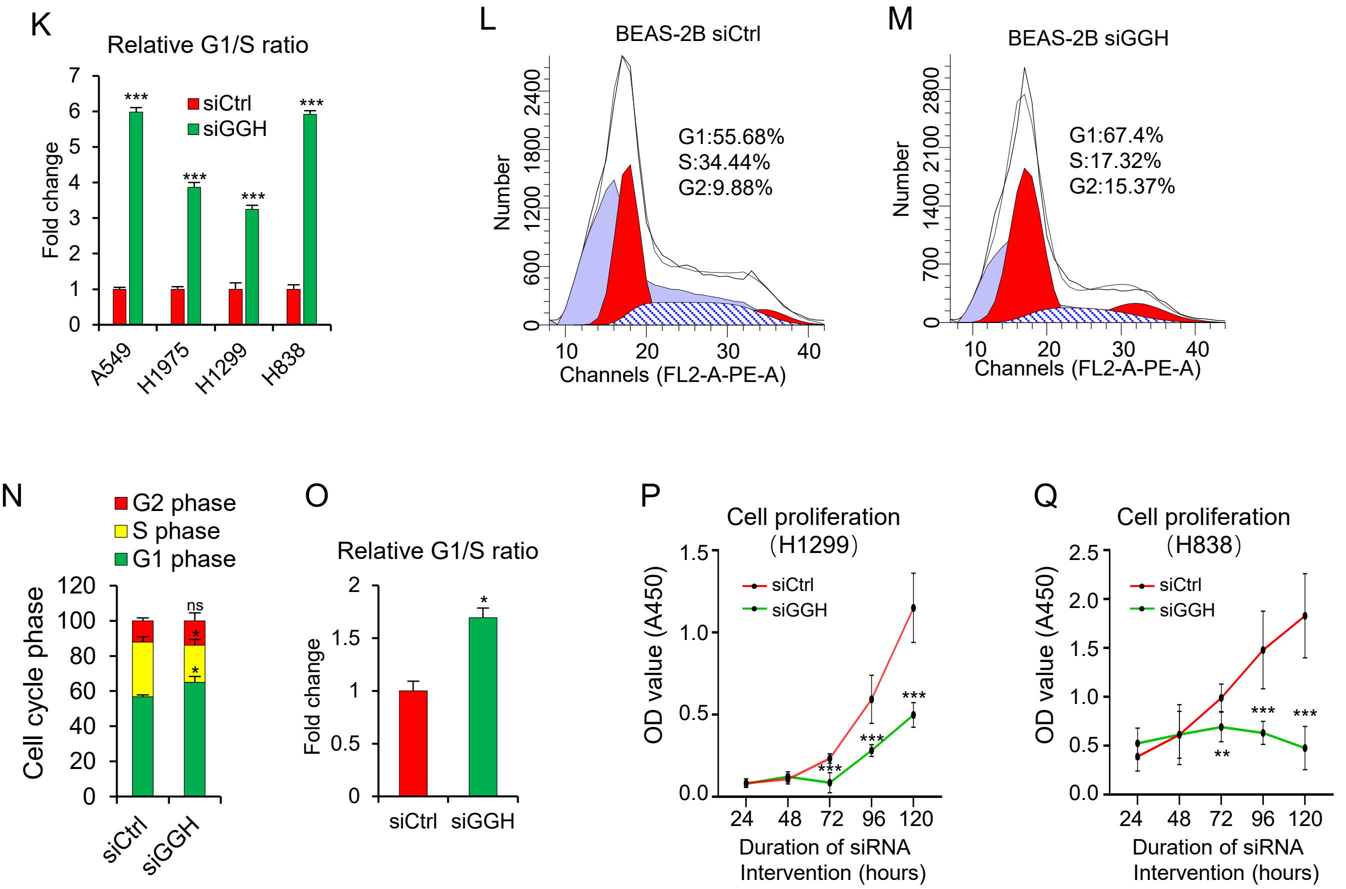
K
Relative G1/S ratio
Fold change
siCtrl
siGGH
***
A549
H1975
H1299
H838
L
BEAS-2B siCtrl
Number
G1:55.68%
S:34.44%
G2:9.88%
Channels (FL2-A-PE-A)
M
BEAS-2B siGGH
Number
G1:67.4%
S:17.32%
G2:15.37%
Channels (FL2-A-PE-A)
N
G2 phase
S phase
G1 phase
Cell cycle phase
ns
siCtrl
siGGH
O
Relative G1/S ratio
Fold change
siCtrl
siGGH
P
Cell proliferation
(H1299)
OD value (A450)
siCtrl
siGGH
Duration of siRNA
Intervention (hours)
Q
Cell proliferation
(H838)
OD value (A450)
siCtrl
siGGH
Duration of siRNA
Intervention (hours)

Figure S1

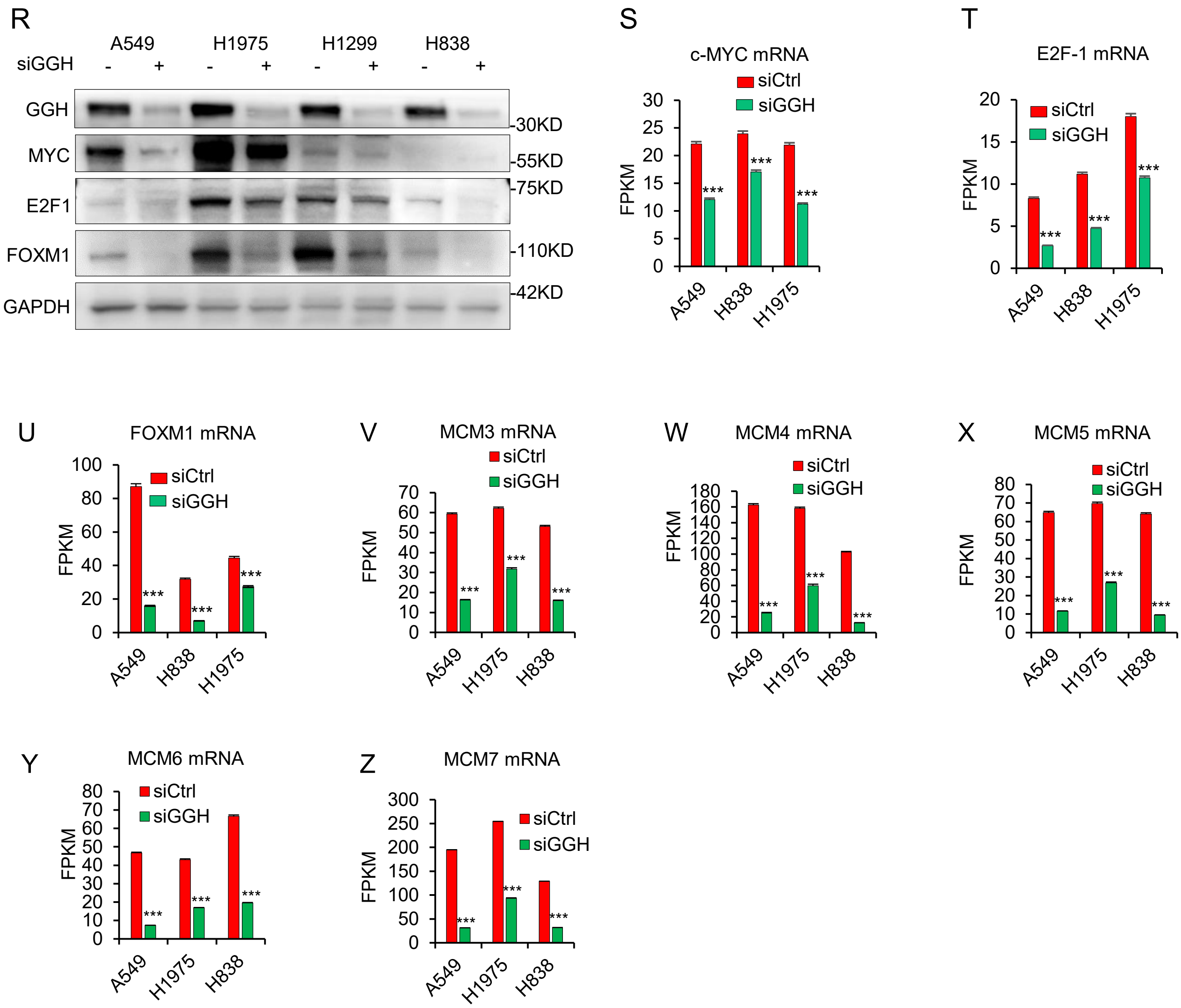

R
A549
H1975
H1299
H838
siGGH
- + - + - + - +
GGH
MYC
E2F1
FOXM1
GAPDH
-30KD
-55KD
-75KD
-110KD
-42KD
S
c-MYC mRNA
siCtrl
siGGH
FPKM
30
25
20
15
10
5
0
***
A549
H838
H1975
T
E2F-1 mRNA
siCtrl
siGGH
FPKM
20
15
10
5
0
***
A549
H838
H1975
U
FOXM1 mRNA
siCtrl
siGGH
FPKM
100
80
60
40
20
0
***
A549
H838
H1975
V
MCM3 mRNA
siCtrl
siGGH
FPKM
70
60
50
40
30
20
10
0
***
A549
H1975
H838
W
MCM4 mRNA
siCtrl
siGGH
FPKM
180
160
140
120
100
80
60
40
20
0
***
A549
H1975
H838
X
MCM5 mRNA
siCtrl
siGGH
FPKM
80
70
60
50
40
30
20
10
0
***
A549
H1975
H838
Y
MCM6 mRNA
siCtrl
siGGH
FPKM
80
70
60
50
40
30
20
10
0
***
A549
H1975
H838
Z
MCM7 mRNA
siCtrl
siGGH
FPKM
300
250
200
150
100
50
0
***
A549
H1975
H838

Figure S1. GGH knockdown impairs the cell cycle and DNA replication. (**A-D**) GGH siRNA knockdown efficiency was measured by qRT-PCR, Western blot, RNA-seq and DIA-MS in NSCLC cells. The 3 siRNAs were pooled for subsequent experiments. Values were mean ± SD. of n =3 independent experiments, *** $p<0.001$. (**E**) Schematic diagram of cell cycle pathway. The networks were built based on the KEGG pathway map: cell cycle (KEGG map04110). The down-regulated genes by GGH silencing were indicated with the red asterisks. (**F**) Heatmap of silencing GGH down-regulated genes of cell cycle pathway in NSCLC cells. (**G**) Schematic diagram of DNA replication pathway. The networks were built based on the KEGG pathway map: DNA replication (KEGG map03030). The down-regulated genes by GGH silencing were indicated with the red asterisks. (**H**) Heatmap of silencing GGH down-regulated genes of DNA replication pathway in NSCLC cells. (**I**) Enriched KEGG pathways of downregulated mRNAs upon GGH knockdown in lung epithelial cell line (BEAS-2B, siGGH/siCtrl<0.65). (**J**) Enriched KEGG pathways of mRNAs positively correlated with GGH in adjacent non-cancerous tissues (r≥0.3, n=156, $p<0.01$). (**K**) The ratio of the G1/S phase upon GGH knockdown in NSCLC cells. Values were mean ± SD from n =3 independent experiments, ***, $p<0.001$. (**L, M**) Representative images of the cell cycle analysis with/without GGH knockdown in lung epithelial cell line (BEAS-2B) using flow cytometry. (**N**) Cell cycle distribution of BEAS-2B cell line with/without GGH knockdown. Values were mean ± SD from n =3 independent experiments, *, $p<0.05$. (**O**) The ratio of the G1/S phase upon GGH knockdown. Values were mean ± SD from n =3 independent experiments, *, $p<0.05$. (**P, Q**) H1299 and H838 cell lines were transfected with siCtrl and siGGH and cultured for 24h, 48h, 72h, 96h and 120h. Cell proliferation was detected by CCK8 assay. Bars indicate SD, n = 6, *, $p<0.05$; **, $p<0.01$; ***, $p<0.001$. (**R**) Western blot of transcription factors c-MYC, E2F1 and FOXM1. (**S-U**) RNA expression of c-MYC, E2F1 and FOXM1 by RNA-seq. Data are shown as mean ± SD, n = 3, *** $p<0.001$. (**V-Z**) RNA expression of MCM3-7 by RNA-seq. Data are shown as mean ± SD, n = 3, *** $p<0.001$.

Figure S2

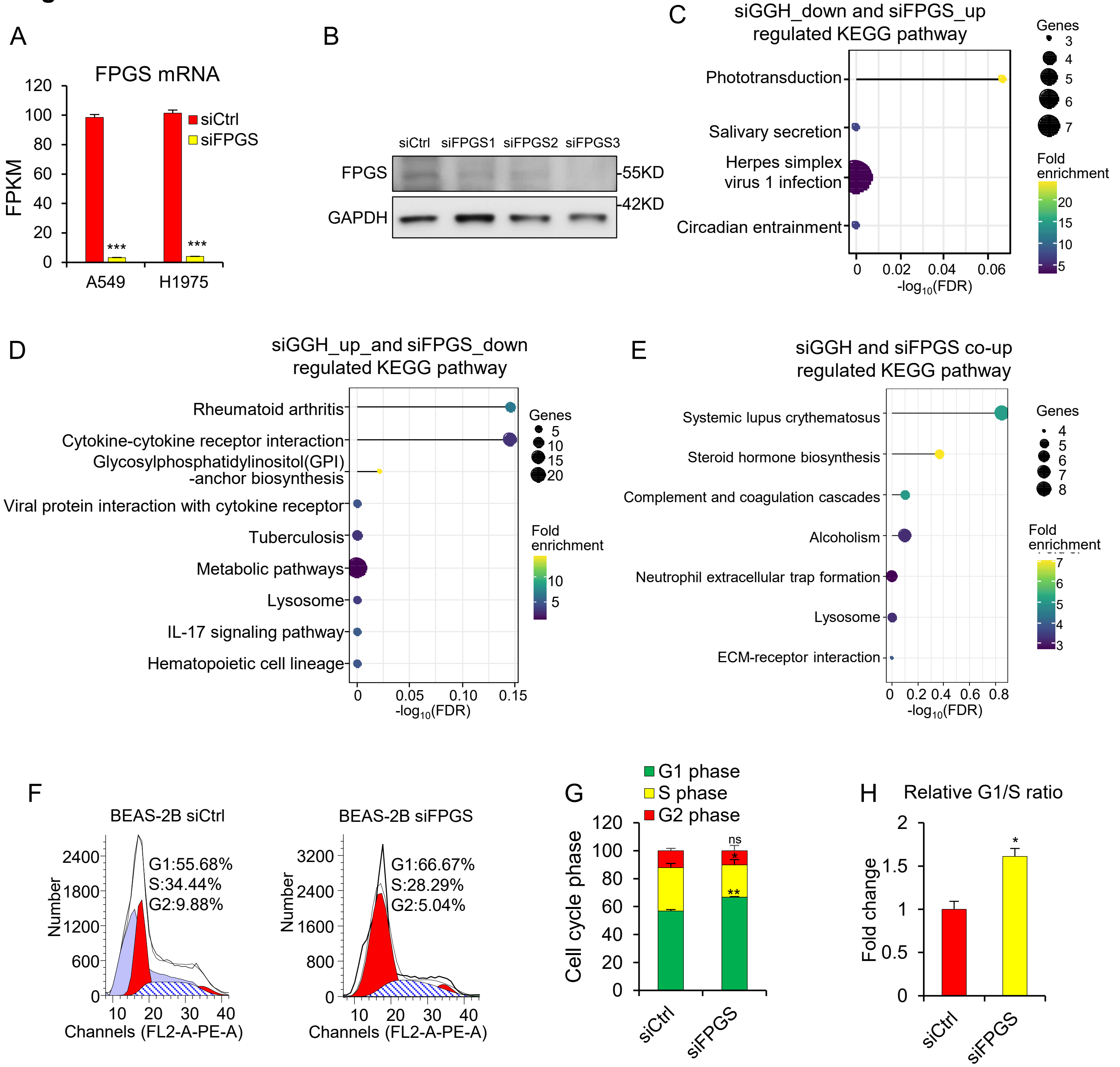

A
FPGS mRNA
FPKM
siCtrl
siFPGS
***
A549
H1975
B
siCtrl
siFPGS1
siFPGS2
siFPGS3
FPGS
GAPDH
55KD
42KD
C
siGGH_down and siFPGS_up regulated KEGG pathway
Phototransduction
Salivary secretion
Herpes simplex virus 1 infection
Circadian entrainment
-log10(FDR)
Genes
Fold enrichment
D
siGGH_up_and siFPGS_down regulated KEGG pathway
Rheumatoid arthritis
Cytokine-cytokine receptor interaction
Glycosylphosphatidylinositol(GPI) -anchor biosynthesis
Viral protein interaction with cytokine receptor
Tuberculosis
Metabolic pathways
Lysosome
IL-17 signaling pathway
Hematopoietic cell lineage
-log10(FDR)
Genes
Fold enrichment
E
siGGH and siFPGS co-up regulated KEGG pathway
Systemic lupus crythematosus
Steroid hormone biosynthesis
Complement and coagulation cascades
Alcoholism
Neutrophil extracellular trap formation
Lysosome
ECM-receptor interaction
-log10(FDR)
Genes
Fold enrichment
F
BEAS-2B siCtrl
G1:55.68%
S:34.44%
G2:9.88%
BEAS-2B siFPGS
G1:66.67%
S:28.29%
G2:5.04%
Number
Channels (FL2-A-PE-A)
G
G1 phase
S phase
G2 phase
Cell cycle phase
ns
*
**
siCtrl
siFPGS
H
Relative G1/S ratio
Fold change
*
siCtrl
siFPGS

Figure S2

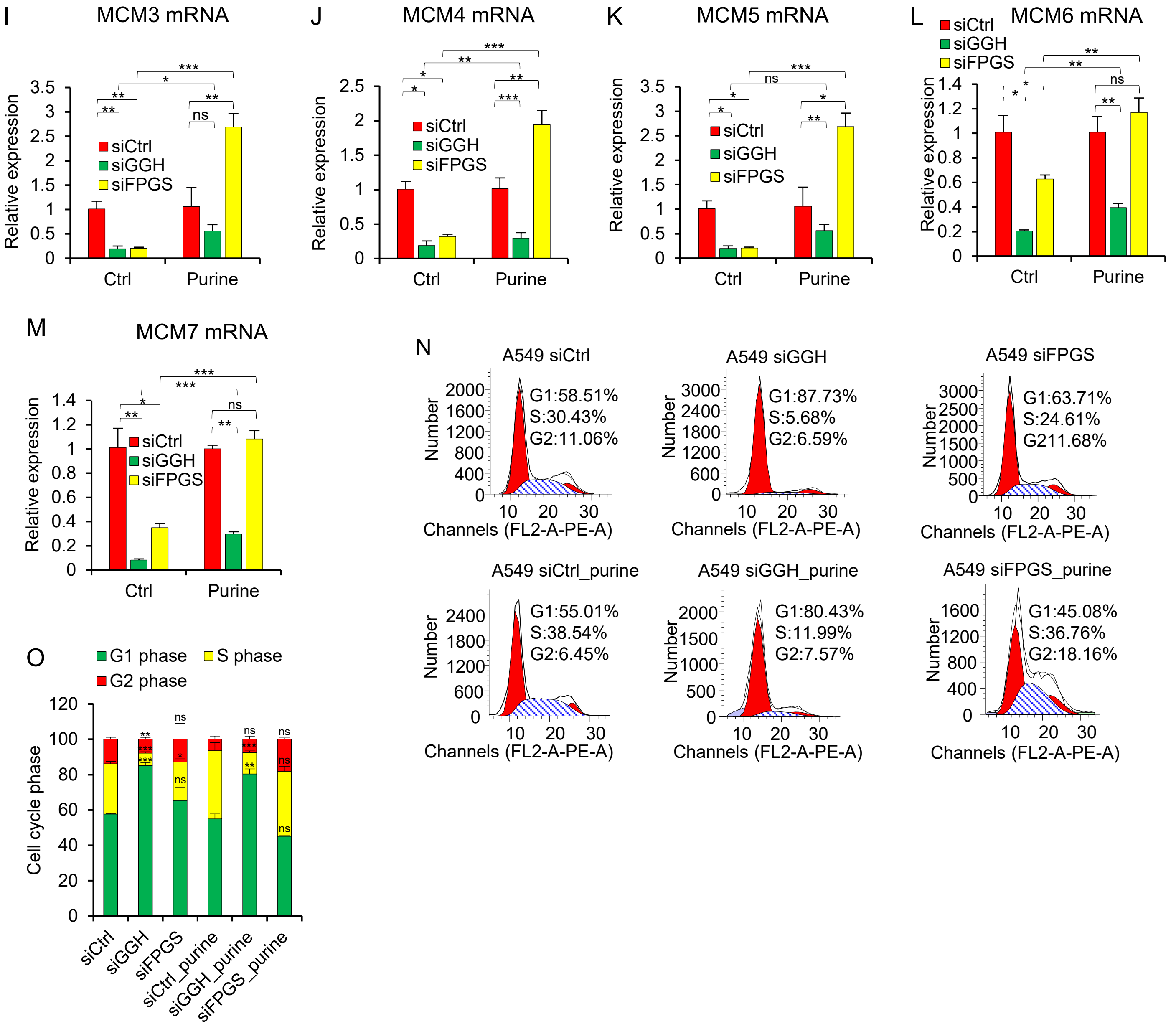

I
MCM3 mRNA
Relative expression
Ctrl
Purine
siCtrl
siGGH
siFPGS
J
MCM4 mRNA
K
MCM5 mRNA
L
MCM6 mRNA
M
MCM7 mRNA
N
A549 siCtrl
G1:58.51%
S:30.43%
G2:11.06%
A549 siGGH
G1:87.73%
S:5.68%
G2:6.59%
A549 siFPGS
G1:63.71%
S:24.61%
G211.68%
A549 siCtrl_purine
G1:55.01%
S:38.54%
G2:6.45%
A549 siGGH_purine
G1:80.43%
S:11.99%
G2:7.57%
A549 siFPGS_purine
G1:45.08%
S:36.76%
G2:18.16%
Number
Channels (FL2-A-PE-A)
O
G1 phase
S phase
G2 phase
Cell cycle phase
siCtrl
siGGH
siFPGS
siCtrl_purine
siGGH_purine
siFPGS_purine

Figure S2

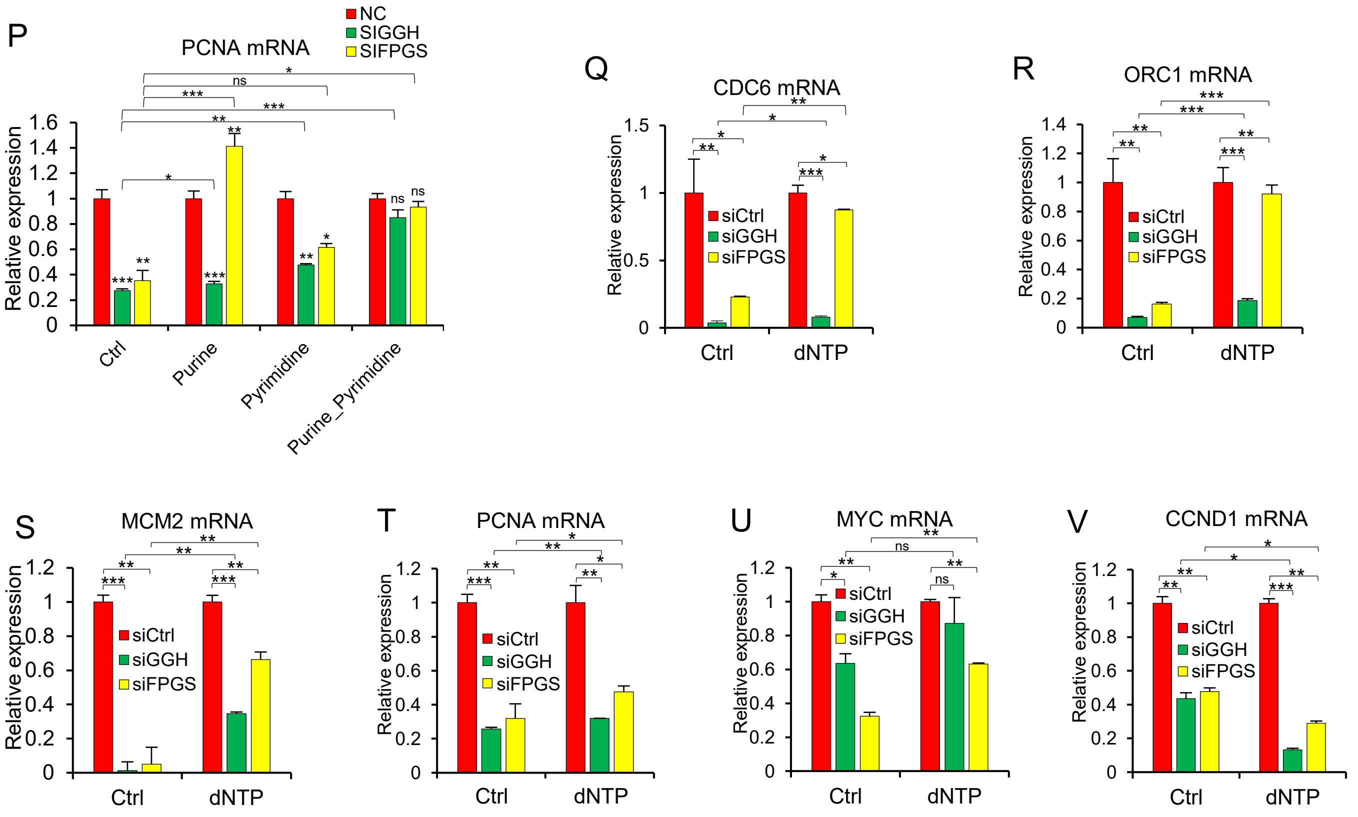
P
PCNA mRNA
NC
SIGGH
SIFPGS
Relative expression
Ctrl
Purine
Pyrimidine
Purine_Pyrimidine
Q
CDC6 mRNA
R
ORC1 mRNA
siCtrl
siGGH
siFPGS
dNTP
S
MCM2 mRNA
T
PCNA mRNA
U
MYC mRNA
V
CCND1 mRNA

Figure S2. GGH silencing diminishes cell cycle and DNA replication independent of folate metabolism pathways. (**A, B**) FPGS siRNA knockdown efficiency was measured by qRT-PCR and Western blot. The 3 siRNAs were pooled for subsequent experiments. Data are shown as mean ± SD, n = 3, *** $p<0.001$. (**C**) Enriched KEGG pathways of the intersection of downregulated mRNAs upon siGGH and upregulated mRNAs upon siFPGS in NSCLC cell lines (siFPGS/siCtrl>1.538 & siGGH/siCtrl<0.65). (**D**) Enriched KEGG pathways of the intersection of upregulated mRNAs upon siGGH and downregulated mRNAs upon siFPGS in NSCLC cell lines (siFPGS/siCtrl<0.65 & siGGH/siCtrl>1.538). (**E**) Enriched KEGG pathways of the intersection of upregulated mRNAs upon siFPGS and siGGH knockdown in NSCLC cell lines (siFPGS/siCtrl>1.538 & siGGH/siCtrl>1.538). (**F**) Representative images of the cell cycle analysis in lung epithelial cell line (BEAS-2B) using flow cytometry. (**G**) Cell cycle distribution of BEAS-2B cell line with/without FPGS knockdown. Values were mean ± SD from n =3 independent experiments, siFPGS vs. siCtrl group, **, $p<0.01$; *, $p<0.05$. (**H**) The ratio of the G1/S phase upon FPGS knockdown in BEAS-2B cells. Values were mean ± SD from n =3 independent experiments, ***, $p<0.001$. (**I-M**) qRT-PCR was used to test the RNA expression of MCM3-7 transfected with either siGGH or siFPGS, and with/without purine treatment. ***, $p<0.001$; **, $p<0.01$; *, $p<0.05$. (**N, O**) Representative images and cell cycle distribution of the cell cycle analysis in NSCLC cell lines after siGGH or siFPGS transfection with/without purine treatment. vs. siCtrl group, ***, $p<0.001$; **, $p<0.01$; *, $p<0.05$. (**P**) qRT-PCR was used to test the RNA expression of PCNA transfected with either siGGH or siFPGS, and with/without purine, pyrimidine, or purine and pyrimidines treatment. Concentration of all regents was 4mM. ***, $p<0.001$; **, $p<0.01$; *, $p<0.05$. (**Q-V**) qRT-PCR was used to test the RNA expression of DNA replication- and cell cycle-related genes transfected with either siGGH or siFPGS, and with/without dNTP treatment. Concentration of dNTP was 2nM. Values were mean±SD from n =3 independent experiments. *, $p<0.05$; **, $p<0.01$; ***, $p<0.001$.

Figure S3

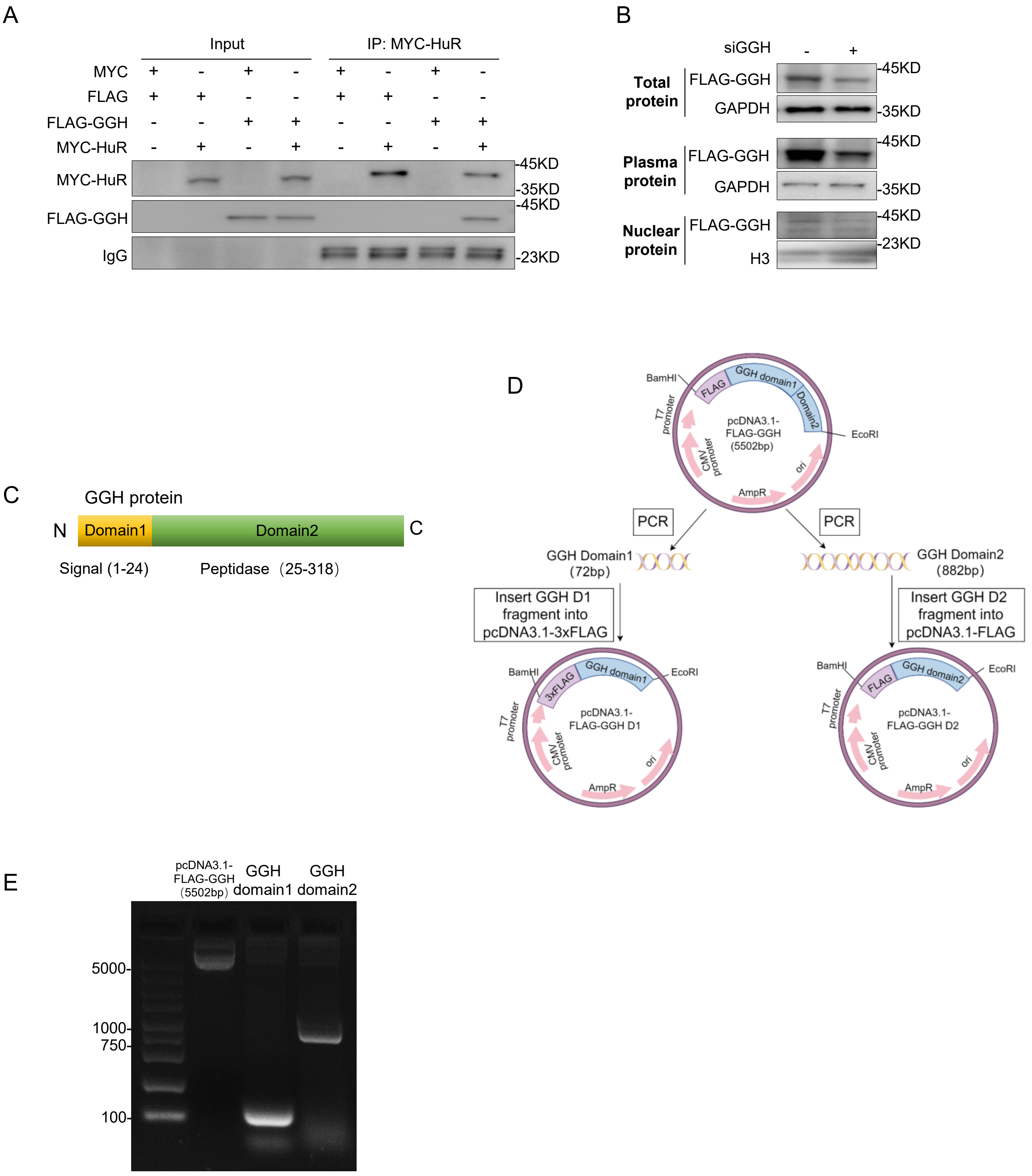

A
Input
IP: MYC-HuR
MYC + - + - + - + -
FLAG + + - - + + - -
FLAG-GGH - - + + - - + +
MYC-HuR - + - + - + - +
MYC-HuR
FLAG-GGH
IgG
-45KD
-35KD
-45KD
-23KD
B
siGGH - +
Total protein
FLAG-GGH
GAPDH
Plasma protein
FLAG-GGH
GAPDH
Nuclear protein
FLAG-GGH
H3
-45KD
-35KD
-45KD
-35KD
-45KD
-23KD
C
GGH protein
N
Domain1
Domain2
C
Signal (1-24)
Peptidase（25-318）
D
BamHI
FLAG
GGH domain1
Domain2
T7 promoter
pcDNA3.1-FLAG-GGH (5502bp)
EcoRI
CMV promoter
ori
AmpR
PCR
PCR
GGH Domain1 (72bp)
GGH Domain2 (882bp)
Insert GGH D1 fragment into pcDNA3.1-3xFLAG
Insert GGH D2 fragment into pcDNA3.1-FLAG
BamHI
3xFLAG
GGH domain1
EcoRI
T7 promoter
pcDNA3.1-FLAG-GGH D1
CMV promoter
ori
AmpR
BamHI
FLAG
GGH domain2
EcoRI
T7 promoter
pcDNA3.1-FLAG-GGH D2
CMV promoter
ori
AmpR
E
pcDNA3.1-FLAG-GGH（5502bp）
GGH domain1
GGH domain2
5000-
1000-
750-
100-

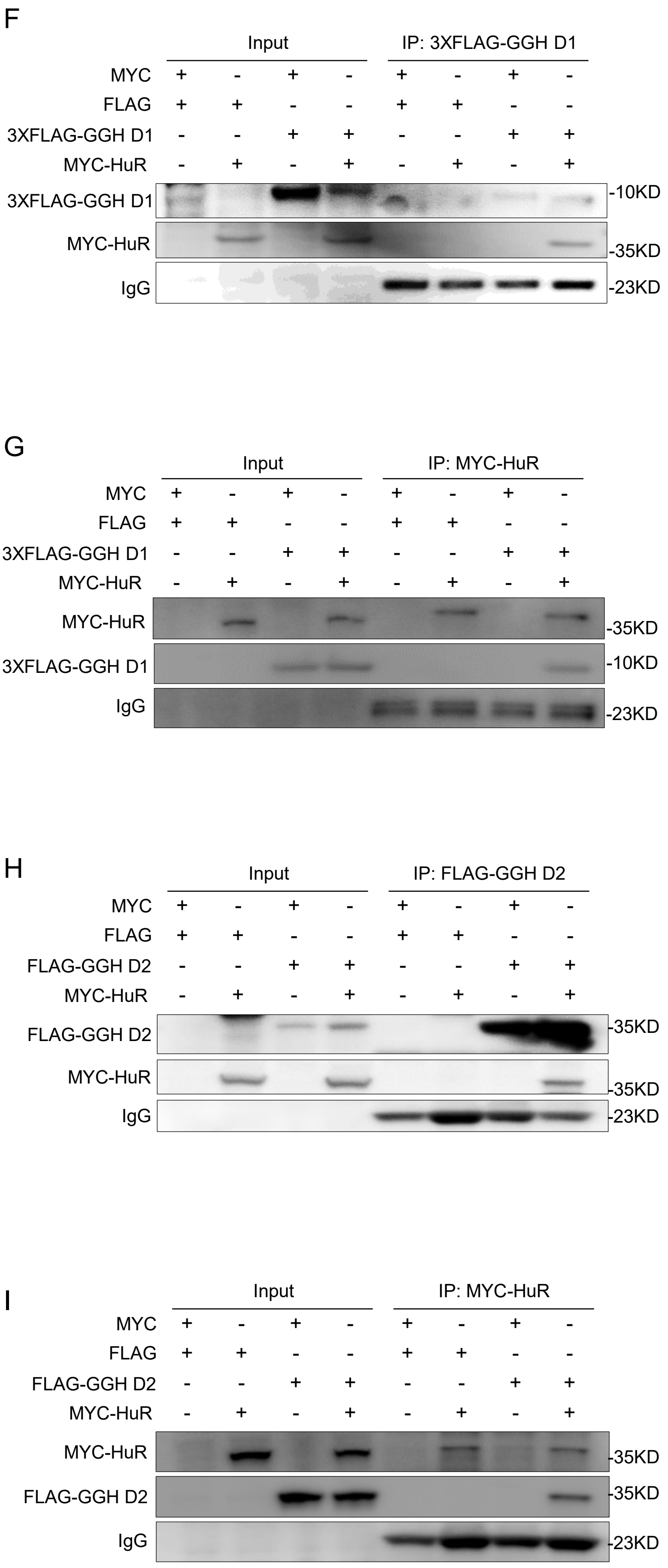
F
Input
IP: 3XFLAG-GGH D1
MYC + - + - + - + -
FLAG + + - - + + - -
3XFLAG-GGH D1 - - + + - - + +
MYC-HuR - + - + - + - +
3XFLAG-GGH D1
MYC-HuR
IgG
-10KD
-35KD
-23KD
G
Input
IP: MYC-HuR
MYC + - + - + - + -
FLAG + + - - + + - -
3XFLAG-GGH D1 - - + + - - + +
MYC-HuR - + - + - + - +
MYC-HuR
3XFLAG-GGH D1
IgG
-35KD
-10KD
-23KD
H
Input
IP: FLAG-GGH D2
MYC + - + - + - + -
FLAG + + - - + + - -
FLAG-GGH D2 - - + + - - + +
MYC-HuR - + - + - + - +
FLAG-GGH D2
MYC-HuR
IgG
-35KD
-35KD
-23KD
I
Input
IP: MYC-HuR
MYC + - + - + - + -
FLAG + + - - + + - -
FLAG-GGH D2 - - + + - - + +
MYC-HuR - + - + - + - +
MYC-HuR
FLAG-GGH D2
IgG
-35KD
-35KD
-23KD

Figure S3

J

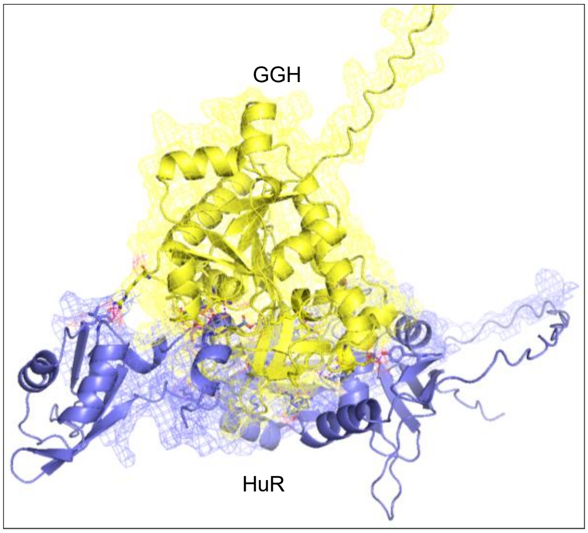

K

HuR residues

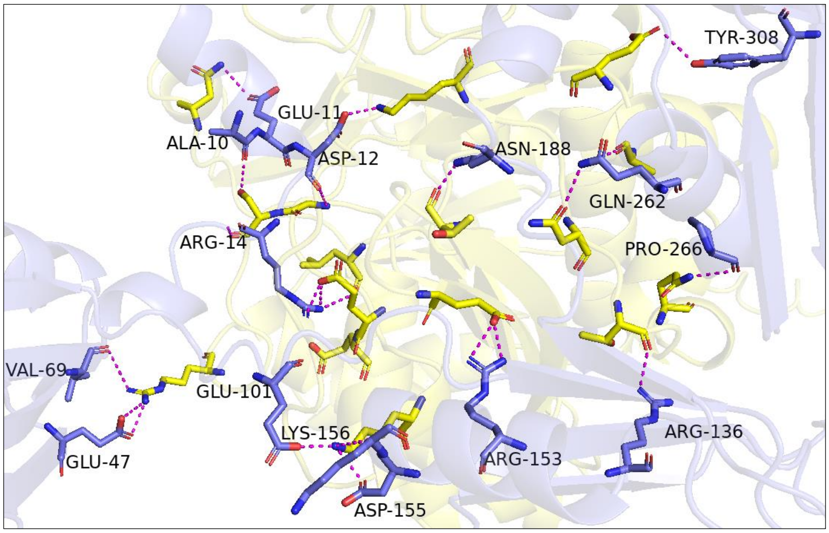

L

GGH residues

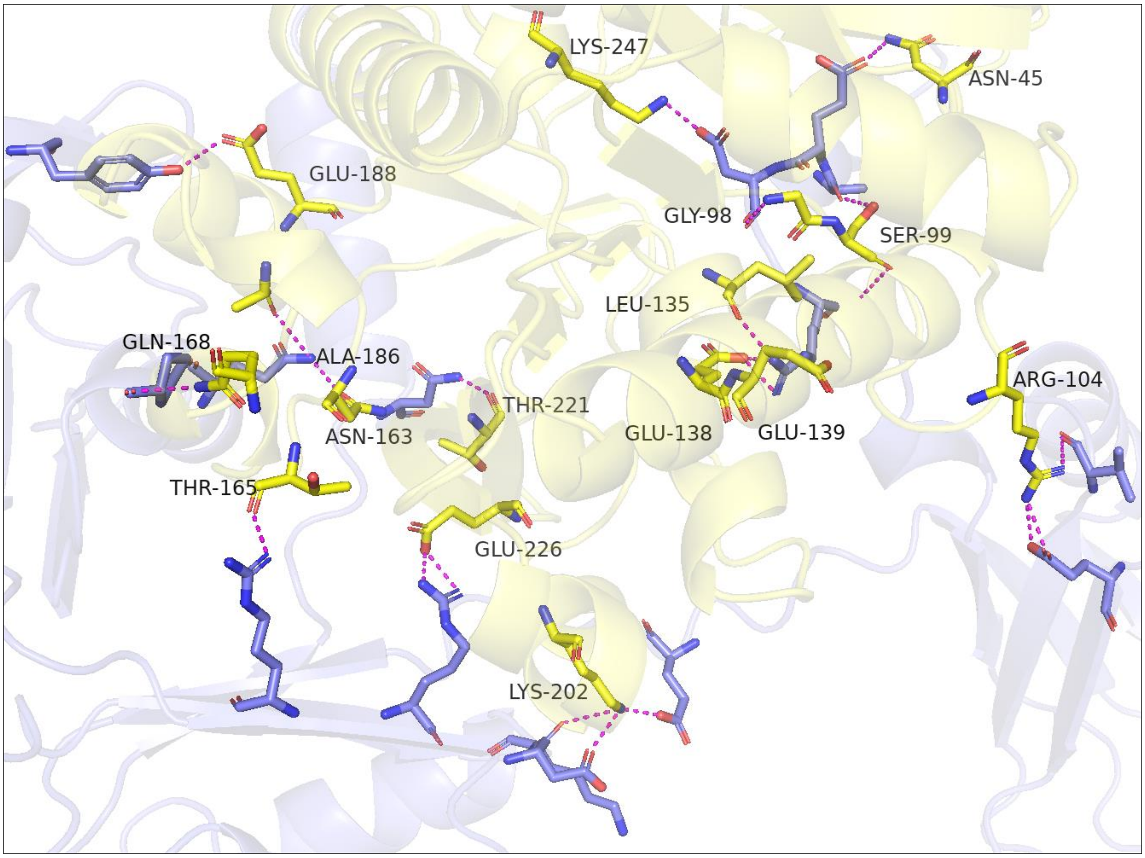

Figure S3

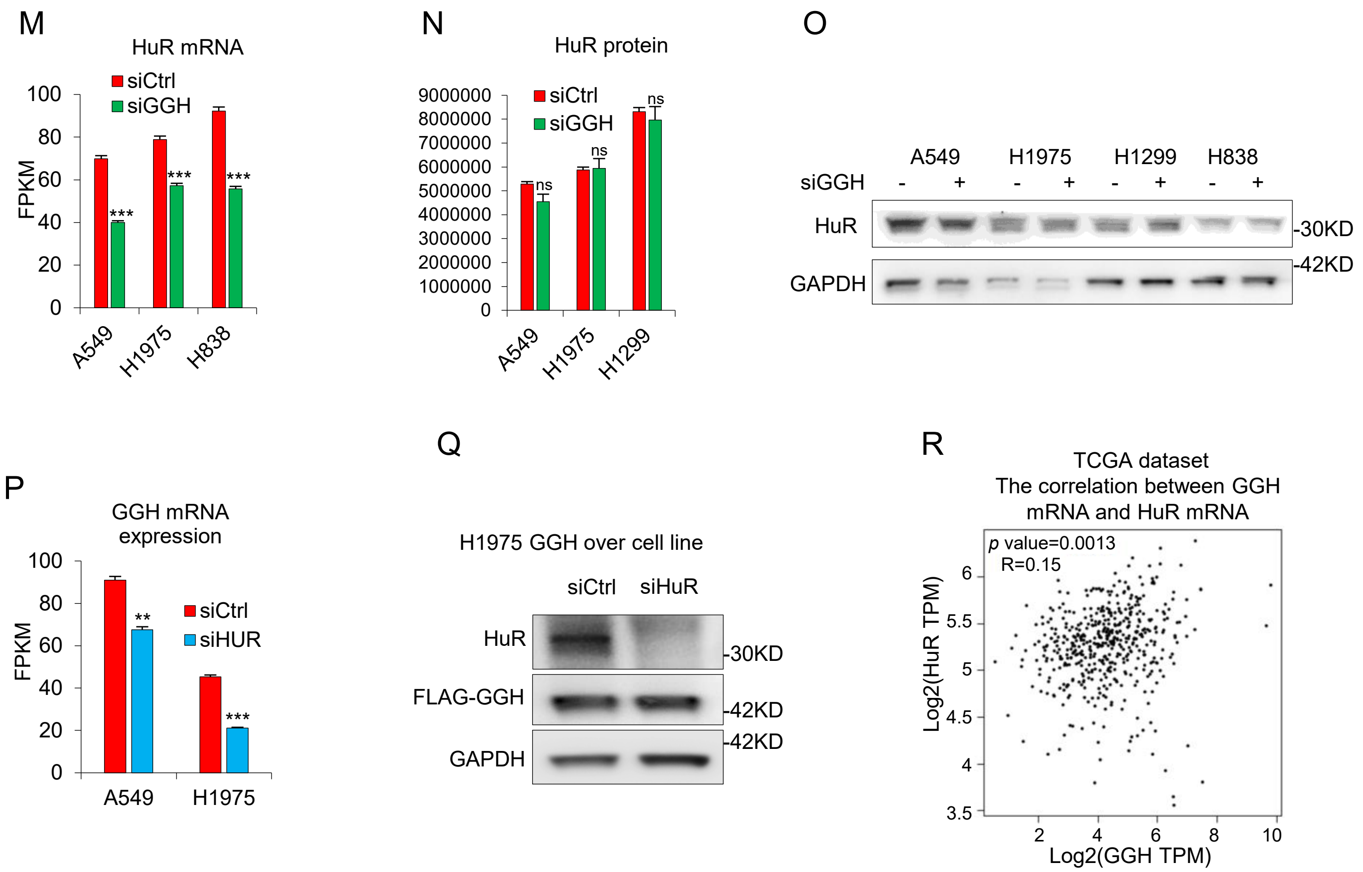

M
HuR mRNA
siCtrl
siGGH
FPKM
100
80
60
40
20
0
***
***
***
A549
H1975
H838
N
HuR protein
siCtrl
siGGH
9000000
8000000
7000000
6000000
5000000
4000000
3000000
2000000
1000000
0
ns
ns
ns
A549
H1975
H1299
O
A549
H1975
H1299
H838
siGGH
- + - + - + - +
HuR
GAPDH
-30KD
-42KD
P
GGH mRNA expression
FPKM
100
80
60
40
20
0
**
***
siCtrl
siHUR
A549
H1975
Q
H1975 GGH over cell line
siCtrl
siHuR
HuR
FLAG-GGH
GAPDH
-30KD
-42KD
-42KD
R
TCGA dataset
The correlation between GGH mRNA and HuR mRNA
p value=0.0013
R=0.15
Log2(HuR TPM)
6
5.5
5
4.5
4
3.5
2
4
6
8
10
Log2(GGH TPM)

Figure S3

S

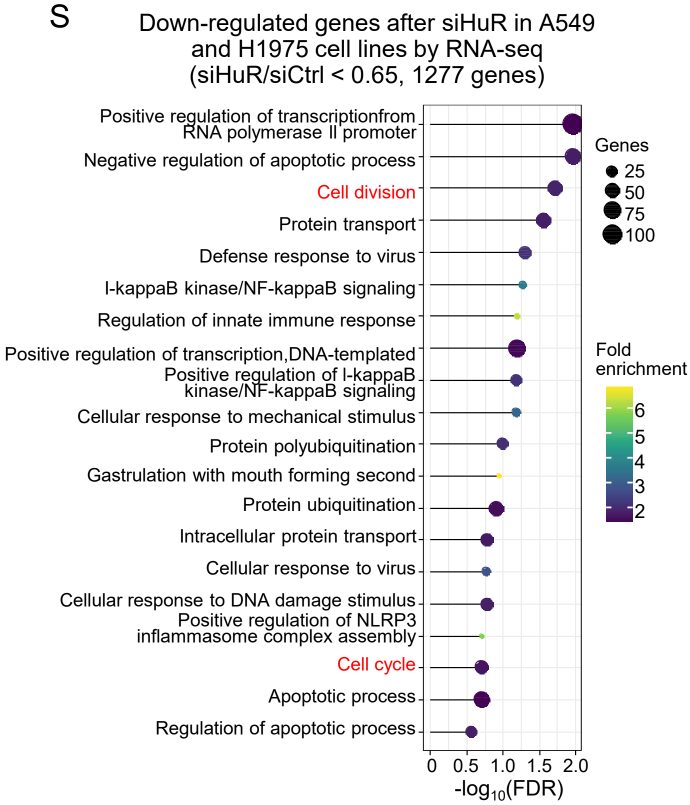

T

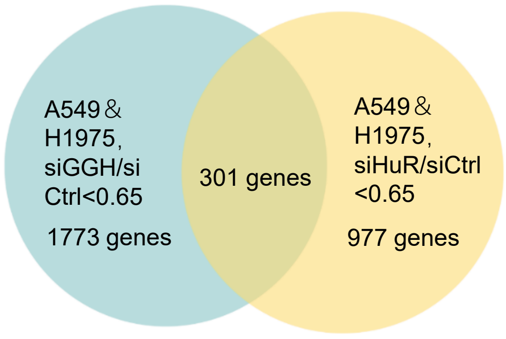

U

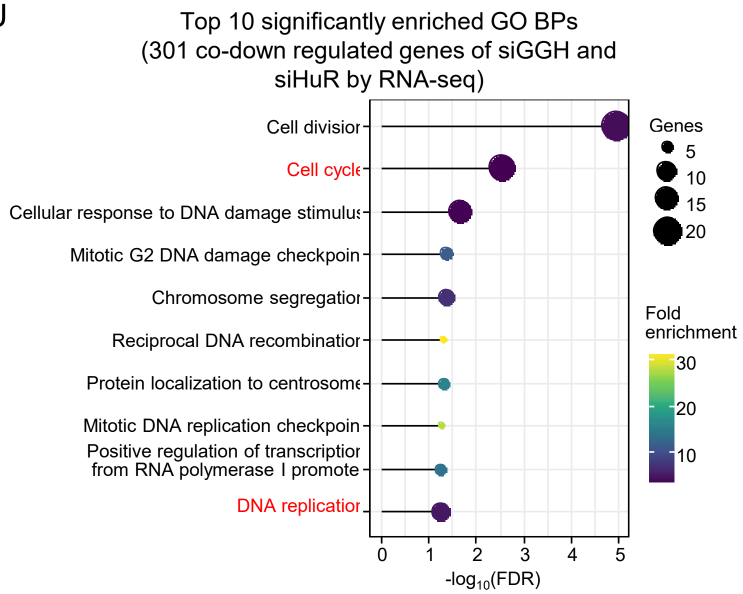

V

Cell division

CCNT1, KIF14, NCAPG, BRCC3, CDC6, MASTL, CDC25C, SKA3, CCSAP, CDC40, SKA1, CENPC, AURKA, SGO2, SYCE2, DSN1, SENP5, CCND1, EPB41L2, CENPJ, NUP43, OIP5, TACC1, FBXO5

Cell cycle

CASP8AP2, MDC1, CCNT1, MCM8, DDIAS, HJURP, BRCA1, BRCC3, MKI67, CDC25C, CCSAP, RCBTB1, AURKA, RBL2, SENP5, EPB41L2, SASS6, NUP43, OIP5

DNA replication

FAM111A, DBF4, RFC1, RMI1, RPA1, RBMS1, RPA2, DTL, TICRR

Figure S3

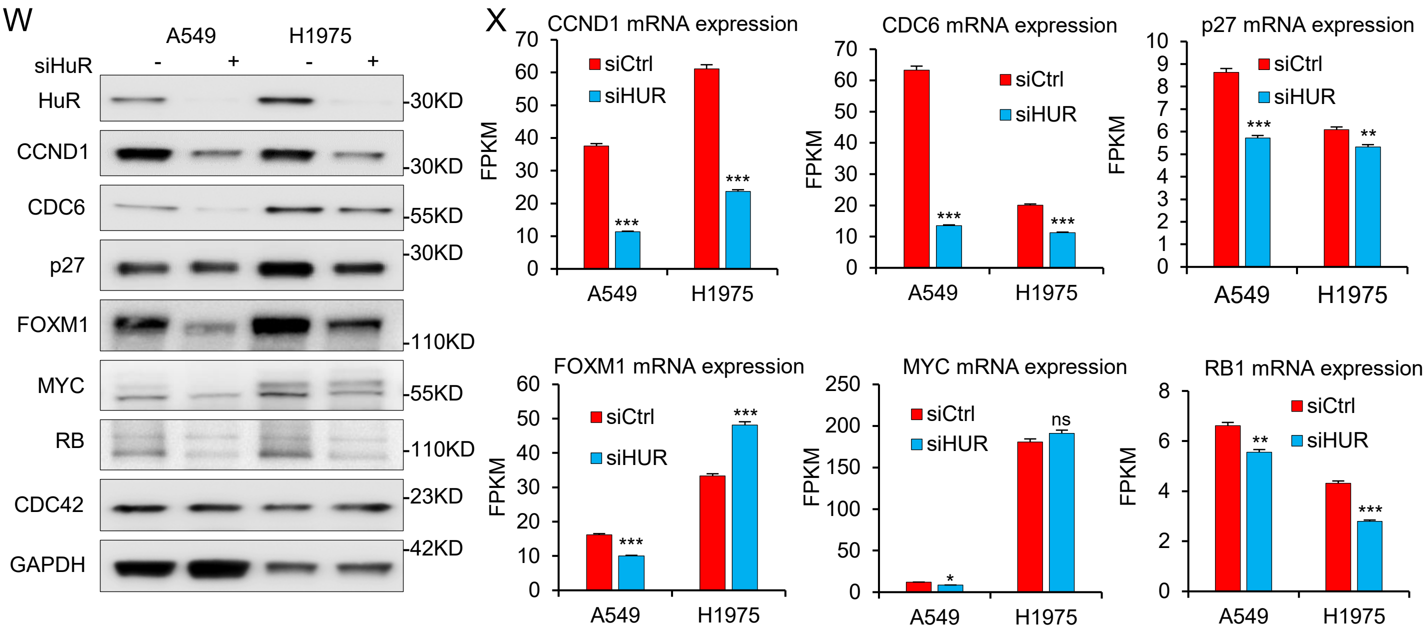

W
A549
H1975
siHuR
- + - +
HuR
CCND1
CDC6
p27
FOXM1
MYC
RB
CDC42
GAPDH
30KD
30KD
55KD
30KD
110KD
55KD
110KD
23KD
42KD
X
CCND1 mRNA expression
CDC6 mRNA expression
p27 mRNA expression
FOXM1 mRNA expression
MYC mRNA expression
RB1 mRNA expression
siCtrl
siHUR
FPKM
A549
H1975
***
**
*
ns

Figure S3. HuR binds with GGH and regulates DNA replication and cell cycle.

(**A**) Co-IP of exogenous HuR shown that HuR precipitates exogenous GGH in 293T cell. (**B**) Total, cytoplasmic and nuclear proteins expression levels of the FLAG-GGH upon GGH knockdown in GGH-overexpressing H1975 cell line. (**C**) The schematic representation of two domains of GGH protein. (**D**) The pcDNA3.1-3XFLAG-GGH domain1 and pcDNA3.1-FLAG-GGH domain 2 (D1 and D2) plasmids were constructed from the pcDNA3.1-FLAG-GGH plasmid. The D1 and D2 fragments were amplified by PCR using the pcDNA3.1-FLAG-GGH plasmid as the template and a set of specific primers. The D1 and D2 amplified fragments are then inserted between the BamHI and EcoRI restriction sites on the pcDNA3.1-3XFLAG or pcDNA3.1-FLAG framework, respectively. (**E**) pcDNA3.1-FLAG-GGH plasmid, D1 and D2 inserted fragments were subjected to electrophoresis. (**F**) Co-IP of exogenous GGH domain1 shown that domain1 precipitated exogenous HuR. (**G**) Co-IP of exogenous HuR shown that HuR precipitated exogenous GGH domain1. (**H**) Co-IP of exogenous GGH domain 2 showed that domain 2 precipitates exogenous HuR. (**I**) Co-IP of exogenous HuR showed that HuR precipitates exogenous GGH domain 2. (**J**) AlphaFold3 database prediction of the modes for GGH (Protein Data Bank (PDB) identification: 1L9X, yellow) binding with HuR (PDB identification: 4FXV, purple). (**K**) Zoom into the image H showing the binding sites of HuR protein. (**L**) Zoom into the image H showing the binding sites of GGH protein. (**M**) RNA expression of HuR after GGH knockdown from RNA-seq. Data are shown as mean ± SD, n = 3, *** p<0.001. (**N**) Protein expression level of HuR after GGH knockdown from DIA-MS. Data are shown as mean ± SD, n = 3. (**O**) Western blot of HuR after GGH knockdown. (**P**) RNA expression of GGH after HuR knockdown from RNA-seq. Data are shown as mean ± SD, n = 3, ** p<0.01, *** p<0.001. (**Q**) Western blot of FLAG-GGH after HuR knockdown in GGH-overexpression H1975 cell line. (**R**) Correlation between mRNA expression of GGH and HuR in TCGA database. (**S**) Enriched KEGG pathways of downregulated mRNAs upon HuR knockdown in A549 and H1975 cell lines (1277 downregulated genes, siHuR/siCtrl<0.65). (**T-V**) Enriched GO BPs of the intersection of downregulated mRNAs upon HuR and GGH knockdown in NSCLC cell lines (301 downregulated genes, siHuR/siCtrl<0.65＆siGGH/siCtrl<0.65). (**W, X**) Western blot and RNA-seq of DNA replication- and cell cycle-related genes. RNA-seq data are shown as mean ± SD, n = 3, * p<0.05, ** p<0.01, *** p<0.001.

Figure S4

A

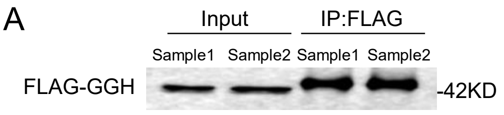

B

ChIP Peaks over Chromosome

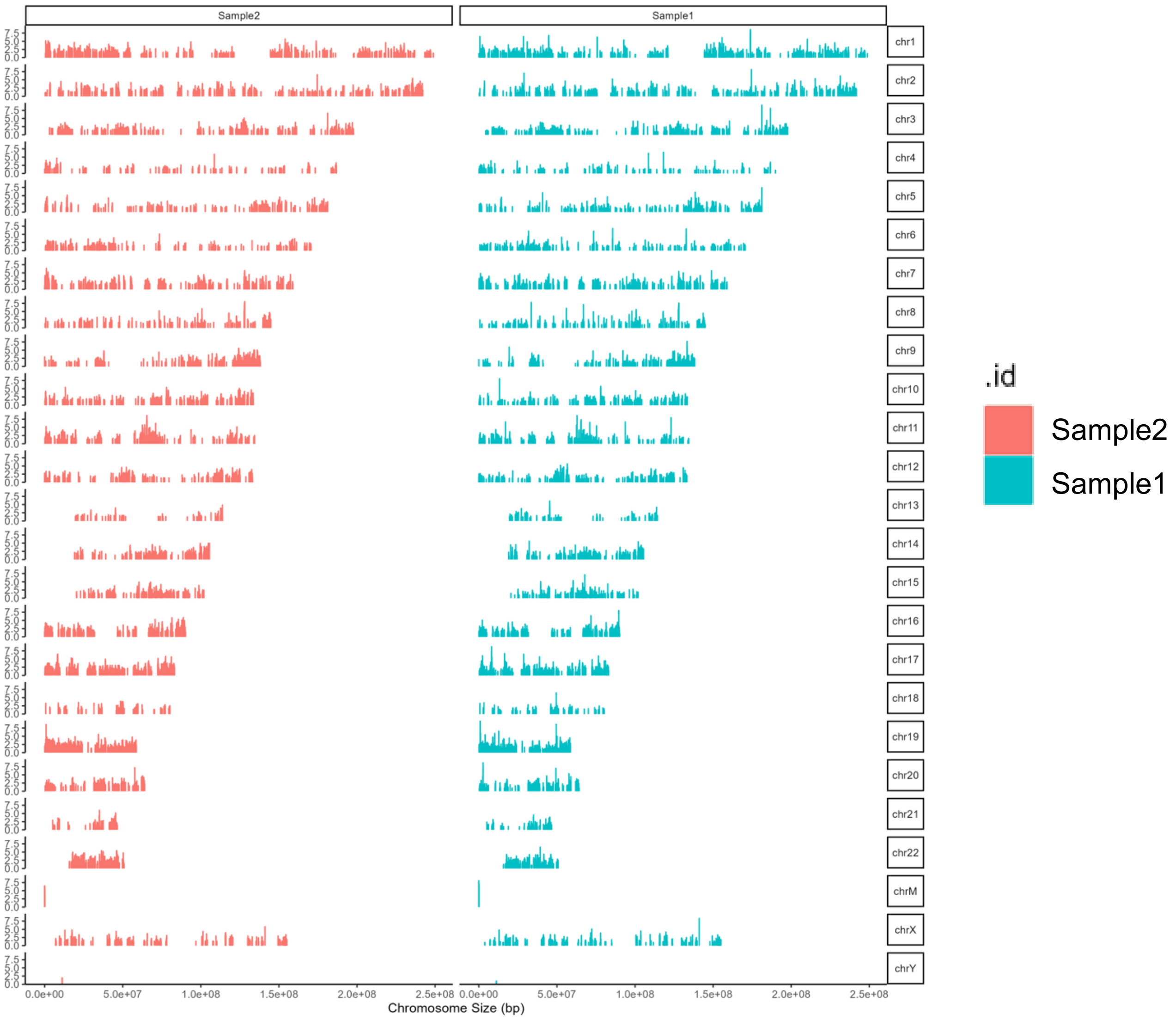

C

Top 10 significantly enriched KEGG pathway of GGH binding with mRNAs (sample2)

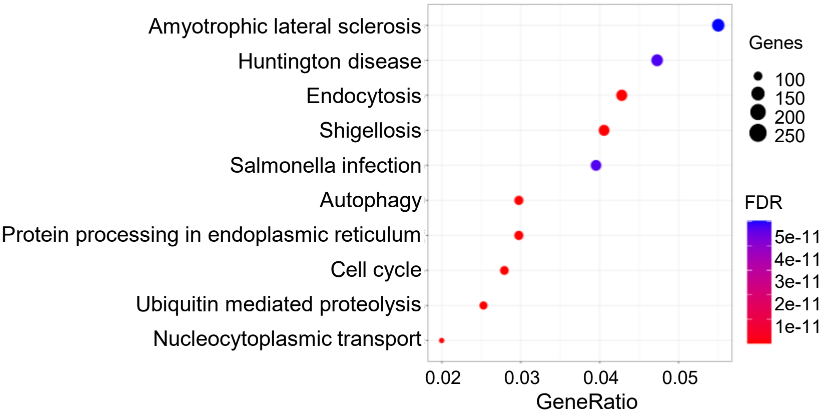

D

CCND1 5'UTR
chr11: 69641236-69641247
GGGAGCGCGG**GGCAGCAGAAGC**GAGAGCCG

CDC6 5'UTR
chr17: 40288043-40288054
CUGGGGUCUG**UGAGGCAGCGGA**GCUGGGUG

MYC 5'UTR
chr8:127736093-127736104
CGCGUGGCGU**GGCGGUGGGCGC**GCAGUGCG

E2F1 5'UTR
chr20: 33686363-33686374
GGACUUUGCA**GGCAGCGGCGGC**CGGGGGCG

PCNA 5'UTR
chr20: 5119819-51198230
CUUCCCGCCU**GCCUGUAGCGGC**GUUGUUGC

Figure S4

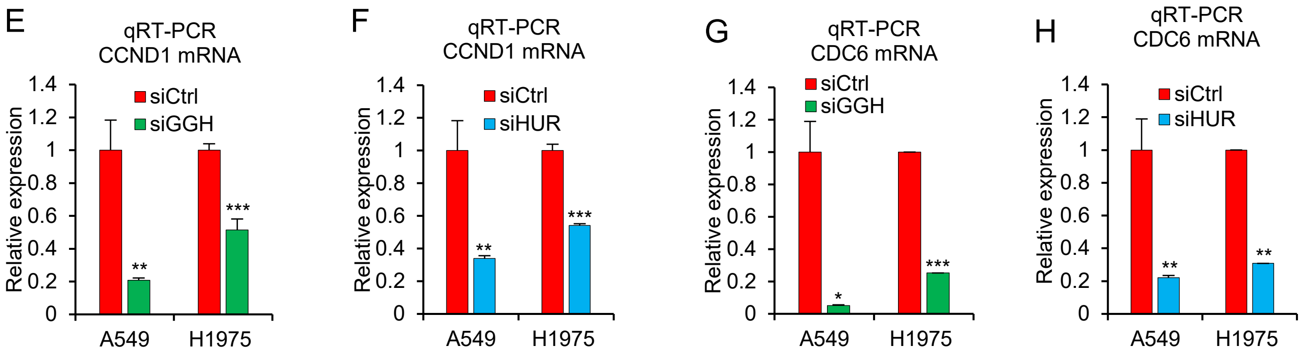
E
qRT-PCR
CCND1 mRNA
siCtrl
siGGH
Relative expression
1.4
1.2
1
0.8
0.6
0.4
0.2
0
**
***
A549
H1975
F
qRT-PCR
CCND1 mRNA
siCtrl
siHUR
Relative expression
**
***
A549
H1975
G
qRT-PCR
CDC6 mRNA
siCtrl
siGGH
Relative expression
*
***
A549
H1975
H
qRT-PCR
CDC6 mRNA
siCtrl
siHUR
Relative expression
**
**
A549
H1975

Figure S4

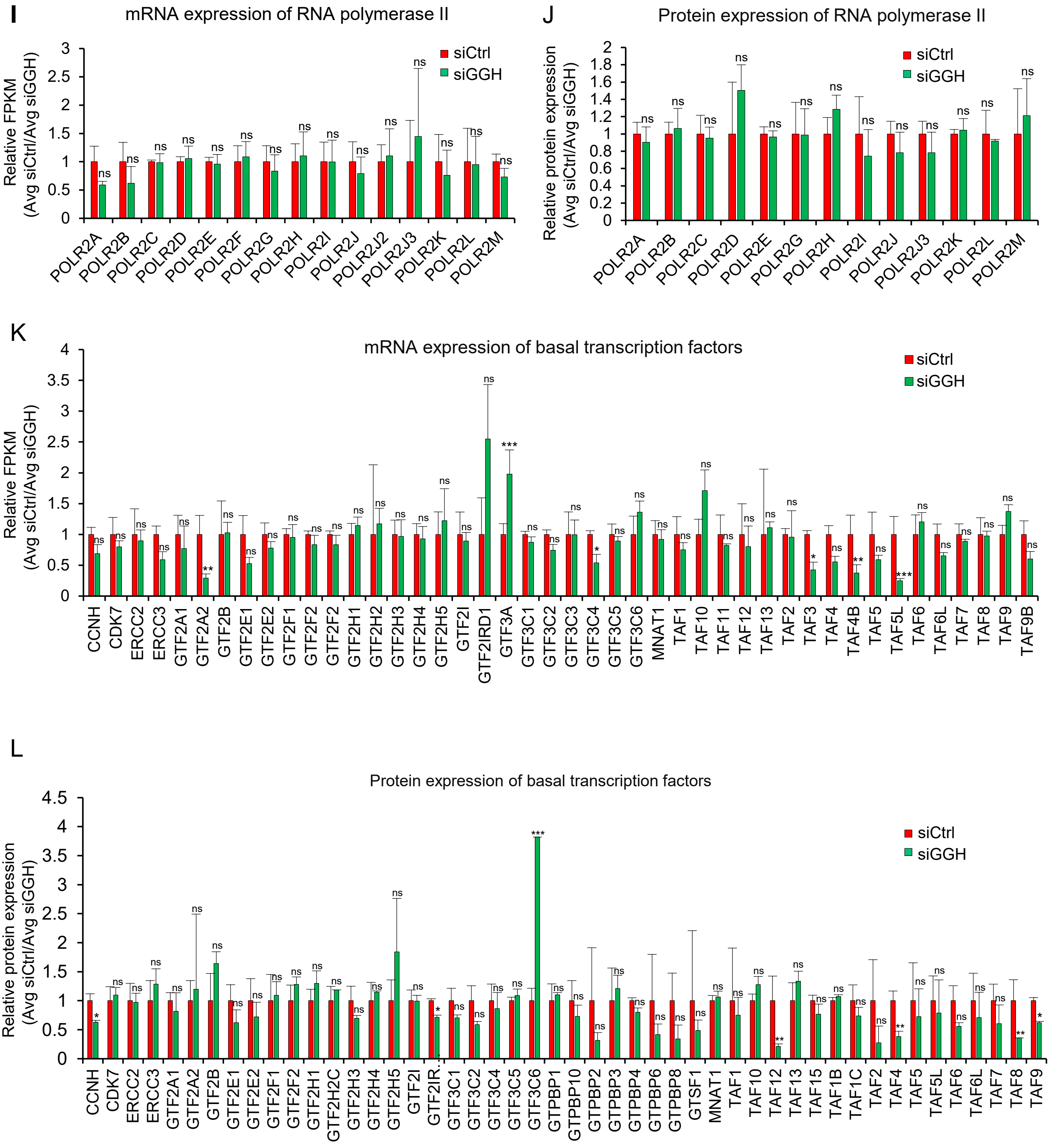
I
mRNA expression of RNA polymerase II
Relative FPKM
(Avg siCtrl/Avg siGGH)
siCtrl
siGGH
POLR2A POLR2B POLR2C POLR2D POLR2E POLR2F POLR2G POLR2H POLR2I POLR2J POLR2J2 POLR2J3 POLR2K POLR2L POLR2M
J
Protein expression of RNA polymerase II
Relative protein expression
(Avg siCtrl/Avg siGGH)
siCtrl
siGGH
POLR2A POLR2B POLR2C POLR2D POLR2E POLR2G POLR2H POLR2I POLR2J POLR2J3 POLR2K POLR2L POLR2M
K
mRNA expression of basal transcription factors
Relative FPKM
(Avg siCtrl/Avg siGGH)
siCtrl
siGGH
CCNH CDK7 ERCC2 ERCC3 GTF2A1 GTF2A2 GTF2B GTF2E1 GTF2E2 GTF2F1 GTF2F2 GTF2F2 GTF2H1 GTF2H2 GTF2H3 GTF2H4 GTF2H5 GTF2I GTF2IRD1 GTF3A GTF3C1 GTF3C2 GTF3C3 GTF3C4 GTF3C5 GTF3C6 MNAT1 TAF1 TAF10 TAF11 TAF12 TAF13 TAF2 TAF3 TAF4 TAF4B TAF5 TAF5L TAF6 TAF6L TAF7 TAF8 TAF9 TAF9B
L
Protein expression of basal transcription factors
Relative protein expression
(Avg siCtrl/Avg siGGH)
siCtrl
siGGH
CCNH CDK7 ERCC2 ERCC3 GTF2A1 GTF2A2 GTF2B GTF2E1 GTF2E2 GTF2F1 GTF2F2 GTF2H1 GTF2H2C GTF2H3 GTF2H4 GTF2H5 GTF2I GTF2IR... GTF3C1 GTF3C2 GTF3C4 GTF3C5 GTF3C6 GTPBP1 GTPBP10 GTPBP2 GTPBP3 GTPBP4 GTPBP6 GTPBP8 GTSF1 MNAT1 TAF1 TAF10 TAF12 TAF13 TAF15 TAF1B TAF1C TAF2 TAF4 TAF5 TAF5L TAF6 TAF6L TAF7 TAF8 TAF9

Figure S4

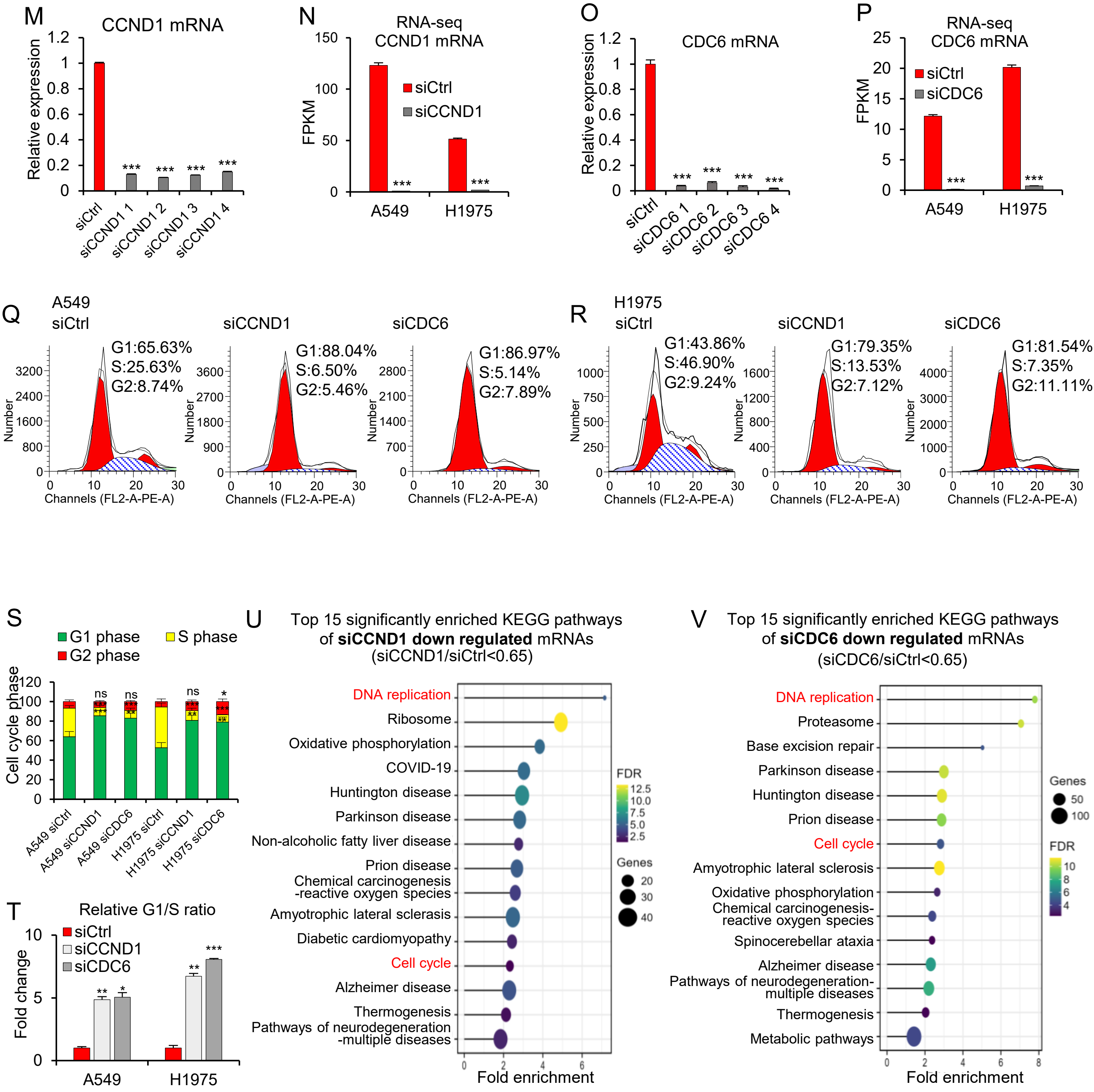
M
CCND1 mRNA
Relative expression
1.2
1
0.8
0.6
0.4
0.2
0
***
siCtrl
siCCND1 1
siCCND1 2
siCCND1 3
siCCND1 4
N
RNA-seq
CCND1 mRNA
FPKM
150
100
50
siCtrl
siCCND1
A549
H1975
O
CDC6 mRNA
siCDC6 1
siCDC6 2
siCDC6 3
siCDC6 4
P
RNA-seq
CDC6 mRNA
25
20
15
10
5
siCDC6
Q
A549
siCtrl
siCCND1
siCDC6
G1:65.63%
S:25.63%
G2:8.74%
G1:88.04%
S:6.50%
G2:5.46%
G1:86.97%
S:5.14%
G2:7.89%
Number
Channels (FL2-A-PE-A)
R
H1975
G1:43.86%
S:46.90%
G2:9.24%
G1:79.35%
S:13.53%
G2:7.12%
G1:81.54%
S:7.35%
G2:11.11%
S
G1 phase
S phase
G2 phase
Cell cycle phase
ns
*
A549 siCtrl
A549 siCCND1
A549 siCDC6
H1975 siCtrl
H1975 siCCND1
H1975 siCDC6
T
Relative G1/S ratio
Fold change
siCtrl
siCCND1
siCDC6
**
U
Top 15 significantly enriched KEGG pathways
of **siCCND1 down regulated** mRNAs
(siCCND1/siCtrl<0.65)
DNA replication
Ribosome
Oxidative phosphorylation
COVID-19
Huntington disease
Parkinson disease
Non-alcoholic fatty liver disease
Prion disease
Chemical carcinogenesis
-reactive oxygen species
Amyotrophic lateral sclerasis
Diabetic cardiomyopathy
Cell cycle
Alzheimer disease
Thermogenesis
Pathways of neurodegeneration
-multiple diseases
FDR
12.5
10.0
7.5
5.0
2.5
Genes
20
30
40
Fold enrichment
V
Top 15 significantly enriched KEGG pathways
of **siCDC6 down regulated** mRNAs
(siCDC6/siCtrl<0.65)
DNA replication
Proteasome
Base excision repair
Parkinson disease
Huntington disease
Prion disease
Cell cycle
Amyotrophic lateral sclerosis
Oxidative phosphorylation
Chemical carcinogenesis-
reactive oxygen species
Spinocerebellar ataxia
Alzheimer disease
Pathways of neurodegeneration-
multiple diseases
Thermogenesis
Metabolic pathways
Genes
50
100
FDR
10
8
6
4
Fold enrichment

Figure S4.GGH binds to RNA directly.

(**A**) Western blot of FLAG-GGH IP in eCLIP assay of two replicates. (**B**) Distribution of GGH-RNA interactions by genome region. (**C**) Enriched KEGG pathways of GGH binding mRNAs of sample 2. (**D**) The presence of a GGH-bound motif in the 5'UTR of multiple 5'UTR mRNAs of cell cycle and DNA replication pathways. (**E-H**) RNA expression levels of CCND1 and CDC6, after transfection with either siGGH or siHuR, measured by qRT-PCR. qRT-PCR and RNA-seq data are both shown as mean ± SD, n = 3, * $p<0.05$, ** $p<0.01$, *** $p<0.001$. (**I-L**) RNA and protein expression levels of RNA polymerase Ⅱ and basal transcription factors, following transfected with siGGH, measured by RNA-seq or DIA-MS. The RNA-seq data presented in our manuscript includes mRNA expression levels in A549, H1975, and H838 cell lines after GGH knockdown. The DIA-MS data presented in our manuscript includes protein expression levels in A549, H1975, and H1299 cell lines after GGH knockdown. These data were generated from these three biological replicates (each comprising three different cell lines), with three technical replicates performed for each cell line. Statistical significance was calculated using DESeq2. Data are shown as mean ± SD, n = 3, * $p<0.05$, ** $p<0.01$, *** $p<0.001$. (**M**) CCND1 siRNAs knockdown efficiency measured by qRT-PCR. The 4 siRNAs were pooled for subsequent experiments. (**N**) CCND1 mixed siRNA knockdown efficiency measured by RNA-seq. Data are shown as mean ± SD, n = 3, *** $p<0.001$. (**O**) CDC6 siRNAs knockdown efficiency, measured by qRT-PCR. The 4 siRNAs were pooled for subsequent experiments. (**P**) CDC6 mixed siRNA knockdown efficiency measured by RNA-seq. Data are shown as mean ± SD, n = 3, *** $p<0.001$. (**Q**) Representative images of the cell cycle analysis in A549 cell line with/without CCND1 or CDC6 knockdown using flow cytometry. (**R**) Representative images of the cell cycle analysis in H1975 cell line with/without CCND1 or CDC6 knockdown using flow cytometry. (**S**) Cell cycle distribution of NSCLC cells with/without CCND1 or CDC6 knockdown. Values were mean ± SD from n=3 independent experiments, ** $p<0.01$, *** $p<0.001$. (**T**) The ratio of the G1/S phase upon FPGS knockdown. Values were mean ± SD from n=3 independent experiments, * $p<0.05$, ** $p<0.01$, *** $p<0.001$. (**U, V**) Enriched KEGG pathways of downregulated mRNAs upon CCND1 or CDC6 knockdown in A549 and H1975 cell lines (siCCND1/siCtrl<0.65 or siCDC6/siCtrl<0.65).

Figure S5

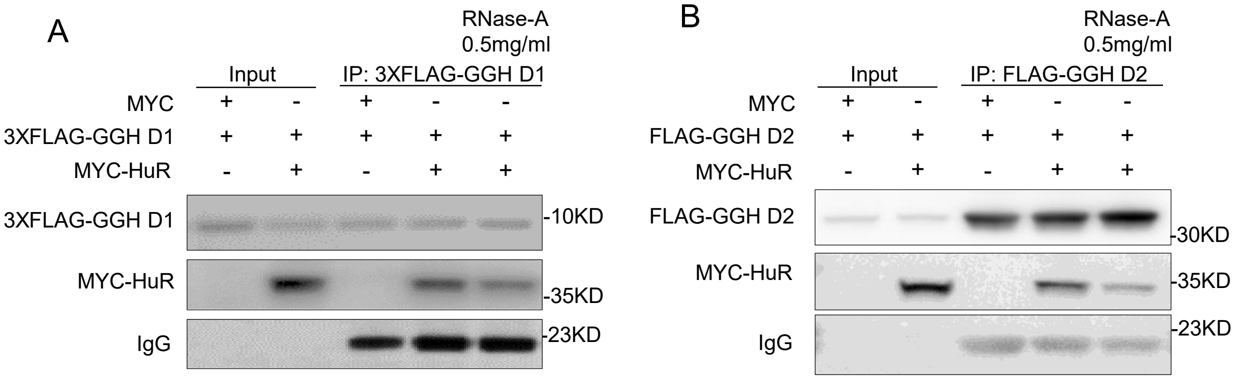

Figure S5. GGH Domain1 and 2 binding to HuR were RNA-dependent.

(**A**) Co-IP assays of 3XFLAG-GGH D1 with or without RNase-A treatment in 293T cell line. Immunoglobulin G (IgG) was used as a negative control. (**B**) Co-IP assays of FLAG-GGH D2 with or without RNase-A treatment in 293T cell line. Immunoglobulin G (IgG) was used as a negative control.

Figure S6

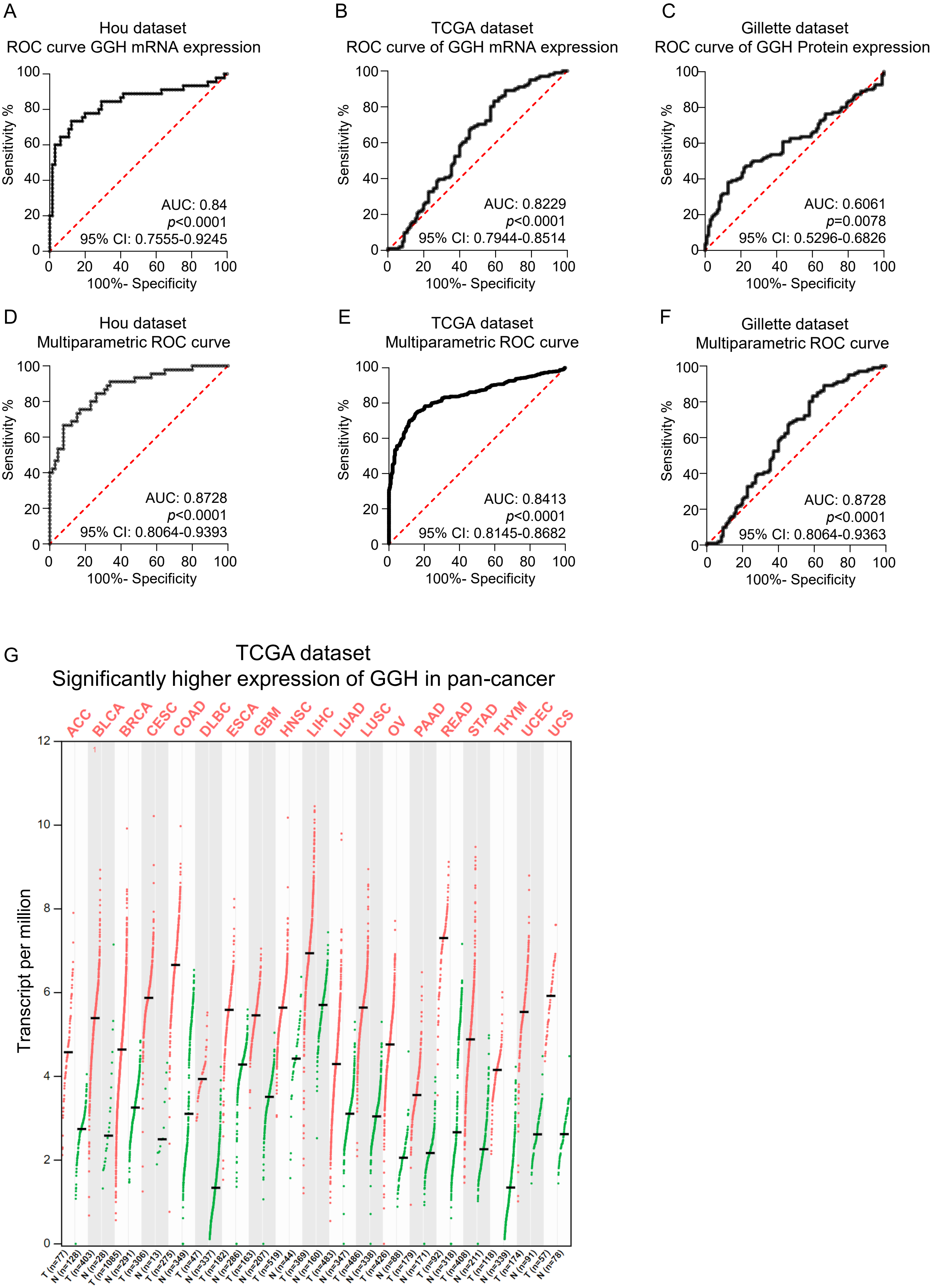

A
Hou dataset
ROC curve GGH mRNA expression
Sensitivity %
100%- Specificity
AUC: 0.84
p<0.0001
95% CI: 0.7555-0.9245
B
TCGA dataset
ROC curve of GGH mRNA expression
Sensitivity %
100%- Specificity
AUC: 0.8229
p<0.0001
95% CI: 0.7944-0.8514
C
Gillette dataset
ROC curve of GGH Protein expression
Sensitivity %
100%- Specificity
AUC: 0.6061
p=0.0078
95% CI: 0.5296-0.6826
D
Hou dataset
Multiparametric ROC curve
Sensitivity %
100%- Specificity
AUC: 0.8728
p<0.0001
95% CI: 0.8064-0.9393
E
TCGA dataset
Multiparametric ROC curve
Sensitivity %
100%- Specificity
AUC: 0.8413
p<0.0001
95% CI: 0.8145-0.8682
F
Gillette dataset
Multiparametric ROC curve
Sensitivity %
100%- Specificity
AUC: 0.8728
p<0.0001
95% CI: 0.8064-0.9363
G
TCGA dataset
Significantly higher expression of GGH in pan-cancer
ACC BLCA BRCA CESC COAD DLBC ESCA GBM HNSC LIHC LUAD LUSC OV PAAD READ STAD THYM UCEC UCS
Transcript per million
T (n=77) N (n=128) T (n=403) N (n=28) T (n=1085) N (n=291) T (n=306) N (n=13) T (n=275) N (n=349) T (n=47) N (n=337) T (n=182) N (n=286) T (n=163) N (n=207) T (n=519) N (n=44) T (n=369) N (n=160) T (n=483) N (n=347) T (n=486) N (n=338) T (n=426) N (n=88) T (n=179) N (n=171) T (n=92) N (n=318) T (n=408) N (n=211) T (n=118) N (n=339) T (n=174) N (n=91) T (n=57) N (n=78)

Figure S6. GGH had significant LUAD diagnostic utility and played a crucial role in pan-cancer. (**A**) Univariate ROC analysis of GGH mRNA expression for distinguishing LUAD vs. normal tissues in the Hou cohort. (**B**) Univariate ROC analysis of GGH mRNA expression in the TCGA LUAD cohort. (**C**) Univariate ROC analysis of GGH protein expression in the Gillette cohort. (**D**) Multiparametric ROC curve from multivariable logistic regression (Hou cohort) with sample type (LUAD/normal) as outcome and GGH mRNA + gender as predictors. (**E**) Multiparametric ROC curve (TCGA cohort) for the model incorporating GGH mRNA + gender. (**F**) Multiparametric ROC curve (Gillette cohort) for the model integrating GGH protein + smoking status + age + gender. (**G**) In TCGA data, GGH mRNA expression was significantly higher in pan-cancer.
